# A new organic molecular probe as a powerful tool for fluorescence imaging and biological study of lipid droplets

**DOI:** 10.7150/thno.79052

**Published:** 2023-01-01

**Authors:** Ri Zhou, Chenguang Wang, Xishuang Liang, Fangmeng Liu, Peng Sun, Xu Yan, Xiaoteng Jia, Xiaomin Liu, Yue Wang, Geyu Lu

**Affiliations:** 1 State Key Laboratory of Integrated Optoelectronics, Key Laboratory of Advanced Gas Sensors of Jilin Province, College of Electronic Science & Engineering, Jilin University, 2699 Qianjin Street, Changchun 130012, China; 2 State Key Laboratory of Supramolecular Structure and Materials, College of Chemistry, Jilin University, 2699 Qianjin Street, Changchun 130012, China; 3 International Center of Future Science, Jilin University, 2699 Qianjin Street, Changchun 130012, China

**Keywords:** fluorescence imaging, live-cell imaging, STED super-resolution imaging, fluorescent probe, lipid droplets

## Abstract

**Background:** Lipid droplets (LDs) are critical organelles associated with many physiological processes in eukaryotic cells. To visualize and study LDs, fluorescence imaging techniques including the confocal imaging as well as the emerging super-resolution imaging of stimulated emission depletion (STED), have been regarded as the most useful methods. However, directly limited by the availability of advanced LDs fluorescent probes, the performances of LDs fluorescence imaging are increasingly unsatisfied with respect to the fast research progress of LDs.

**Methods:** We herein newly developed a superior LDs fluorescent probe named **Lipi-QA** as a powerful tool for LDs fluorescence imaging and biological study. Colocalization imaging of** Lipi-QA** and LDs fluorescent probe Ph-Red was conducted in four cell lines. The LDs staining selectivity and the photostability of **Lipi-QA** were also evaluated by comparing with the commercial LDs probe Nile Red. The in-situ fluorescence lifetime of **Lipi-QA** in LDs was determined by time-gated detection. The cytotoxicity of **Lipi-QA** was assessed by MTT assay. The STED saturation intensity as well as the power- and gate time-dependent resolution were tested by Leica SP8 STED super-resolution nanoscopy. The time-lapse 3D confocal imaging and time-lapse STED super-resolution imaging were then designed to study the complex physiological functions of LDs.

**Results:** Featuring with the advantages of the super-photostability, high LDs selectivity, long fluorescence lifetime and low STED saturation intensity, the fluorescent probe **Lipi-QA** was capable of the long-term time-lapse three-dimensional (3D) confocal imaging to in-situ monitor LDs in 3D space and the time-lapse STED super-resolution imaging (up to 500 STED frames) to track the dynamics of LDs with nanoscale resolution (37 nm).

**Conclusions:** Based on the state-of-the-art fluorescence imaging results, some new biological insights into LDs have been successfully provided. For instance, the long-term time-lapse 3D confocal imaging has surely answered an important and controversial question that the number of LDs would significantly decrease rather than increase upon starvation stimulation; the time-lapse STED super-resolution imaging with the highest resolution has impressively uncovered the fission process of nanoscale LDs for the first time; the starvation-induced change of LDs in size and in speed has been further revealed at nanoscale by the STED super-resolution imaging. All of these results not only highlight the utility of the newly developed fluorescent probe but also significantly promote the biological study of LDs.

## Introduction

Lipid droplets (LDs), which are composed of the neutral lipid cores and the surrounding phospholipid monolayer membranes, are ubiquitous organelles presenting in almost all eukaryotic cells 1,2. LDs are formed by budding off from the endoplasmic reticulum (ER) into the cytoplasm, where the nascent LDs undergo maturation and gradually increase their sizes by fusing with the other LDs or synthesizing additional neutral lipids. Accordingly, LDs are widely varied in size: the diameters are only 30‒60 nm for the nascent LDs and increasing to 0.1-1 μm or even 10 μm for the mature ones. In a long history, LDs were regarded as the inert fat particles that stored the excessive lipids in cells. However, more recent studies have continuously discovered the pivotal roles of LDs in a number of cellular processes including energy homeostasis, lipid metabolism, membrane proteins expression, membranes trafficking and so on 1-5. Consequently, the study of LDs has emerged as one of the most attractive topics of cell biology during the past decade.

To visualize LDs and reveal their versatile functions, the confocal and wide-field fluorescence microscopies have been widely employed with sub-micrometer resolution (~250 nm) 6-10. The emerging super-resolution fluorescence microscopies, such as stimulated emission depletion microscopy (STED) and photoactivated localization microscopy (PALM), successfully broke the resolution limit of light diffraction and make it possible to further visualize the small/nascent LDs with nanoscale resolution 11-16. For example, our group developed a super-photostable LDs fluorescent probe Lipi-DSB for STED super-resolution imaging, successfully visualizing the nanoscale fusion process of nascent LDs 12. Xiao and coworkers designed a series of visible-light-activated fluorescence switch probes for PALM super-resolution imaging of LDs, achieving the localization precision of 13 nm in the LDs images 13. Xu and coworkers reported a hydrogen-bond-sensitive fluorogenic LDs probe LD-FG for structured illumination microscopy (SIM) super-resolution imaging, impressively tracking various dynamic process of cellular LDs 14. Niu and coworkers developed a series of rigidly structured solvatochromic fluorescent probes for SIM super-resolution imaging of cytosolic and nuclear LDs, acquiring the unprecedented spatial resolution for nuclear LDs of 142 nm 15. Obviously, the LDs fluorescence imaging performances in terms of the resolution, the number of frames, the signal-to-background contrast, and so on are dependent on not only the fluorescence microscopies themselves but also the LDs fluorescent probes. Currently, Nile Red and BODIPY 493/503 are the most representative fluorescent probes for confocal imaging of LDs. However, their photostability and LDs-staining specificity are still quite unsatisfied for the long-term time-lapse confocal imaging and three-dimensional (3D) confocal imaging. More importantly, the super-resolution imaging has additional strict requirements for the LDs fluorescent probes. For instance, the STED super-resolution imaging which employs an excitation laser like confocal imaging as well as a special donut-shaped STED laser for depletion has two inherent requirements: (1) the fluorescent probe can be efficiently depleted by the STED laser, which is essential to improve the resolution behind light diffraction; (2) the fluorescent probe should display significantly high photostability, because the STED laser is extremely strong (10^1^‒10^2^ MW cm^‒2^) and the resolution would be continuously improved upon increasing the STED laser intensity 17,18. Furthermore, STED super-resolution imaging with time-gated detection would be able to further improve the resolution, which requires the fluorescent probe has a long fluorescence lifetime 19. While the commonly used fluorescent probes do not satisfy these requirements, STED super-resolution imaging of small/nascent LDs with nanoscale resolution is a highly challenging task 11,12. Therefore, the lack of advanced LDs fluorescent probes directly hampers visualization of LDs with higher resolution as well as more frames (*i.e.* longer time), thus significantly limiting the biological study of LDs.

To solve this issue, we herein would like to present a new fluorescent probe named **Lipi-QA** as a powerful tool for LDs fluorescence imaging. The fluorescent probe features with the advantages of super-photostability, high LDs selectivity, long fluorescence lifetime and low STED saturation intensity. These advantages enable the fluorescent probe to be highly desired for superior confocal imaging as well as STED super-resolution imaging, *e.g.* the long-term time-lapse 3D confocal imaging (about 2000 confocal frames) to in-situ monitor the changing process of LDs in 3D space and the time-lapse STED super-resolution imaging (up to 500 STED frames) to track the dynamics of LDs with nanoscale resolution (37 nm). Consequently, LDs have been impressively visualized at the unprecedented spatial and temporal dimensionality. Moreover, these state-of-the-art fluorescence imaging results have provided some new insights into the biological study of LDs.

## Results

### Molecular design and photophysical property of Lipi-QA

**Figure 1:**
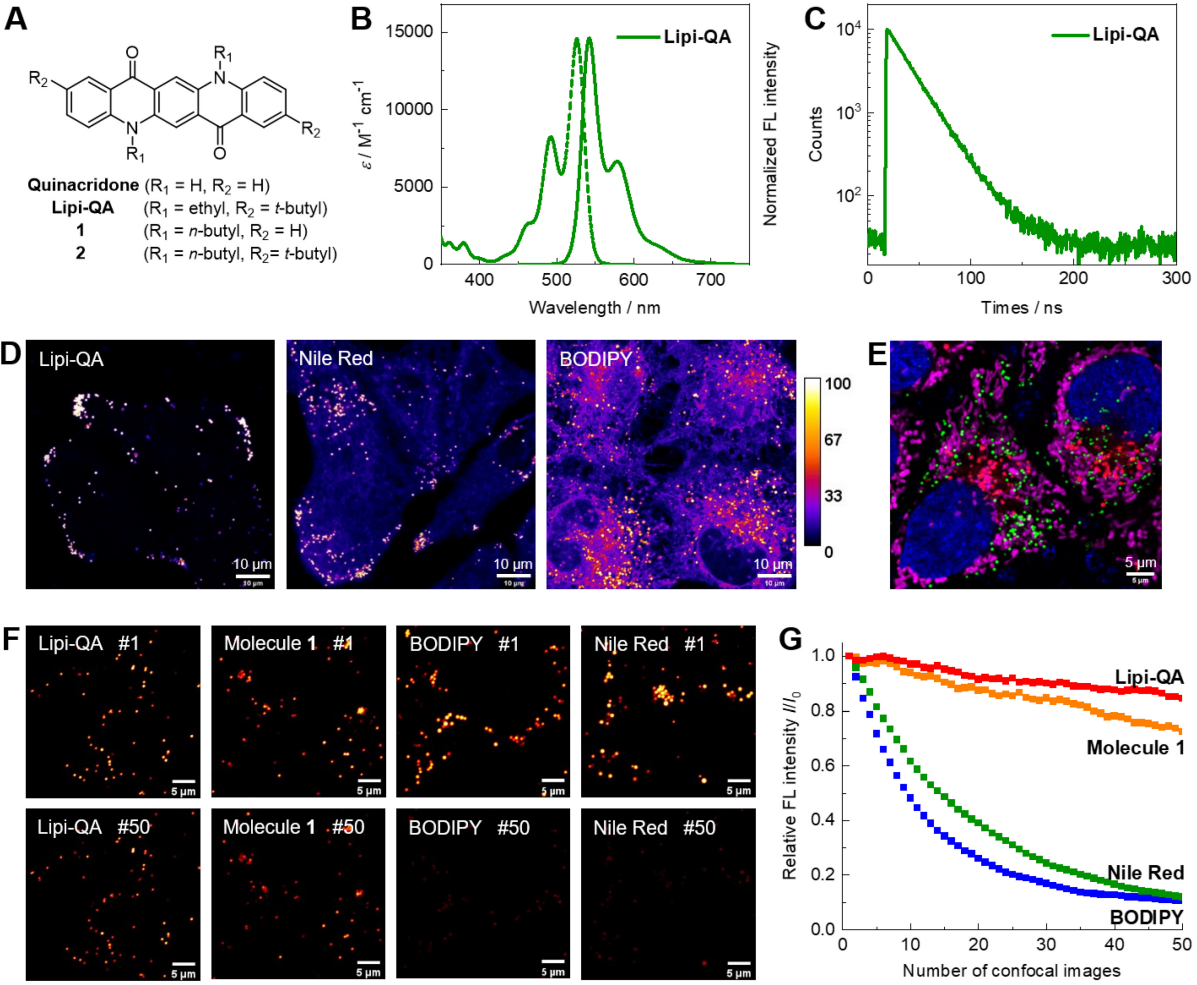
(A) Molecular structure of the LDs fluorescent probe **Lipi-QA** and its analogous molecules **1**‒**2**. (B‒C) Absorption spectrum, fluorescence spectrum and decay profile of **Lipi-QA** in dichloromethane solution. (D) Comparison of the LDs staining specificity between **Lipi-QA**, Nile Red and BODIPY 493/503 in living HepG2 cells with the same staining condition of 2 μM, 2 h. *λ*_ex_ = 488 nm, *λ*_em_ = 500‒700 nm. Scale bar: 10 μm. (E) Multicolor confocal imaging of living HeLa cells labelled with Hoechst 33342 (blue, 20 μM, 0.5 h), **Lipi-QA** (green, 2 μM, 2 h), LysoTracker Red (red, 200 nM, 0.5 h), and MitoTracker Deep Red (pink, 50 nM, 0.5 h). Scale bar: 5 μm. (F‒G) Comparison of the photostability between **Lipi-QA**, molecules **1**, Nile Red, and BODIPY 493/503. The confocal images of HeLa cells stained with these fluorescent probes (2 μM, 2 h) were repeatedly recorded under the identical and intense excitation condition; the confocal images of number 1 and 50 were shown. *λ*_ex_ = 488 nm, *λ*_em_ = 500‒700 nm. Scale bar: 5 μm.

Our design strategy for the generation of highly photostable LDs probe Lipi-QA is based on the famous pigment quinacridone (**Figure 1A**). In more than a half of century, quinacridone and its derivatives are widely used as industrial paints with the characters of exceptional photo- and thermal-stability 20. In recent years, their superior utilities in optoelectronic devices have been also explored,* e.g.* used as highly efficient emitting materials in organic light-emitting diodes 21. Inspiring by the advantages of photostability and efficient emission, we herein envisioned that quinacridone would be a highly attractive π-skeleton for constructing new fluorescent probe. Moreover, the character of long fluorescence lifetime would further largely increase the value of quinacridone-based fluorescent probe for time-gated/lifetime imaging with higher resolution and lower background. Based on quinacridone skeleton, we introduce two alkyl groups on the *N*-atoms as well as two bulky* tert*-butyl moieties on the terminals of molecule with the consideration of tuning LDs specificity, cell-permeability, and photostability. The detailed chemical synthesis of target fluorescent probe **Lipi-QA** and its analogous molecules **1**‒**2** with various alkyl substituents are descripted in the **Supplementary Information**. The molecule structure of **Lipi-QA** with planar conformation has been further revealed by the X-ray single crystal structure analysis (**Figure S1**).

The photophysical property of fluorescent probe **Lipi-QA** was then studied in solutions. Since the absorption and emission spectra are insensitive to the solvent polarities (**Figure S2**), its spectra in dichloromethane solution are chosen for presentation. As shown in **Figure 1B**, **Lipi-QA** displays a structured absorption spectrum with maximum (*λ*_abs_) of 526 nm and molar extinction coefficient (*ε*) of 1.46 × 10^4^ M^-1^ cm^-1^. The emission spectrum of **Lipi-QA** is symmetric to the absorption, reflecting the π→π* transition character in the excited state. The emission (*λ*_em_) maximum is 542 nm and the fluorescence quantum yield (*Φ*_F_) is up to 0.98. The high *Φ*_F_ as well as the narrow emission spectrum are strongly relative to its planar π-skeleton. The emission property of **Lipi-QA** was further investigated in aqueous mixture of PBS/DMSO (V/V = 3/7) as well as in oleic acid. In both of them, **Lipi-QA** displays similar emission (about 560 nm) with the high *Φ*_F_ of ~0.65 (**Figure S2** and **Table S2**). The fluorescence lifetime (*τ*) of **Lipi-QA** is further determined to be 21.8 ns (**Figure 1C**). This value is quite long for organic fluorescent molecules. Even in comparison to the probes ADOTA, CDOTA, KU530 and SeTau-425 which have been specially developed as the long fluorescence lifetime ones for lifetime imaging (**Table S1**), the probe** Lipi-QA** is still a strong competitor in terms of the long fluorescence lifetime as well as the high fluorescence brightness (*ε* × *Φ*_F_). In addition, the photophysical properties of molecules **1**‒**2** have been studied (**Figure S3** and **Table S1**). Similar to **Lipi-QA**, these two molecules display high *Φ*_F_, narrow emission spectra, as well as long fluorescence lifetimes. To gain a deep insight into the character of long fluorescence lifetime, TD-DFT calculation has been conducted (**Figure S4**). The first excited state of **Lipi-QA** corresponding to the transition of HOMO to LUMO features a very small oscillator strength (*f* = 0.069). According to the equations *k*_r_ ∝ *ν*^2^*f* and* τ* = *Φ*_F_/*k*_r_ 22, (where *k*_r_ represents the radiative decay rate constant, and *ν* represents the wavenumber of absorption maximum), a very small value of *f* would provide a significantly decreased *k*_r_ and a large value of *τ* could be thus expected.

### Fluorescence Imaging Property of Lipi-QA

The performance of **Lipi-QA** as a LDs specific fluorescent probe was evaluated by co-staining experiments with various cell lines (HeLa, HepG2, HT22, and U251). Live cells were incubated with this probe **Lipi-QA** (2 μM) and a red-emissive LDs specific fluorescent probe Ph-Red (1 μM) for 2 h 23. Irrespective of the cell types, the green imaging channels of **Lipi-QA** and the red imaging channels of Ph-Red are well overlapped with each other (**Figure S5**). The high Pearson's correlation coefficient (*R*) values of 0.92 to 0.96 reveal the high selectivity of **Lipi-QA** towards LDs. It should be noted that the selectivity of **Lipi-QA** is significantly higher than the representative LDs fluorescent probes Nile Red and BODIPY 493/503. In all of four cell lines, **Lipi-QA** solely labels LDs with high specificity, while Nile Red and BODIPY 493/503 stain plasma membrane and/or cytoplasm to a certain extent (**Figure 1D**, **Figure S6** and** S7**). In addition, the in-situ emission spectra of **Lipi-QA**, Nile Red and BODIPY 493/503 in cellular LDs were also studied (**Figure S8**). The emission maxima are 529 nm, 563 nm and 505 nm, respectively.

The comparison between Lipi-QA and its analogous molecules **1**‒**2** provide some interesting insights regarding the LDs imaging property. The co-staining experiments of molecules **1**‒**2** and Ph-Red demonstrate that molecule **2** (*R* = 0.94) displays high LDs selectivity while molecule **1** (*R* = 0.79) does not work so well (**Figure S9**). Moreover, it is found that the cells stained with molecule **2** exhibit much lower fluorescence signals than that of **Lipi-QA**, although molecule **2** also has high LDs selectivity. These results reveal the critical roles of alkyl groups on tuning the LDs selectivity and cell-permeability of fluorescent probe. The high LDs selectivity combining with the narrow emission spectrum enables **Lipi-QA** to be readily applied in multicolor confocal imaging which is a powerful tool to visualize multiple subcellular structures at the same time. For this experiment, the cellular organelles nucleus, LDs, lysosomes, and mitochondria were labelled with Hoechst 33342, **Lipi-QA**, LysoTracker Red and MitoTracker Deep Red, respectively. Without cross-talk between each imaging channel, the four-color confocal imaging was successfully obtained with desired quality (**Figure 1E** and **Figure S10**).

Importantly, **Lipi-QA** displays outstanding photostability. Under the identical and intense excitation condition (*λ*_ex_ = 488 nm, about 100 times stronger laser power of the common imaging condition), the confocal images of HeLa cells labelled with this probe were repeatedly recorded and compared with the other probes (**Figure 1F‒G** and **Figure S11**). While Nile Red and BODIPY 493/503 were quickly photobleached during repeatedly imaging, **Lipi-QA** and molecule **1** exhibited dramatically higher photostability. After recording 50 confocal images, molecule **1** still maintained 72% of its initial fluorescence signal, which was much higher than Nile Red (12%) and BODIPY 493/503 (11%). Moreover, the photostability of **Lipi-QA** (85%) was even better than that of molecule **1**. This result highlights the outstanding photostability of quinacridone-based π-skeleton, also reveals the significant effect of tert-butyl group on the photostability of fluorescent probe.

Another important feature of **Lipi-QA** is the long fluorescence lifetime (*τ*) in cellular LDs. The value of in-situ fluorescence lifetime was determined using picosecond-pulsed excitation and time-gated detection. After HeLa cells were labelled with **Lipi-QA**, a series of confocal images of the same area were recorded at various delay times, while taking a constant time width for detection window (*Δt* = 3.5 ns) (**Figure S12**). The fluorescence intensities of images were plotted as a function of the delay times. After fitting the plot with a single exponential decay function, the fluorescence lifetime of **Lipi-QA** was determined to be 19.2 ns in cellular LDs. This value was comparable to the probe in dichloromethane solution (*τ* = 21.8 ns) and much longer than Nile Red (4.5 ns) 24 and BODIPY 493/503 (7.2 ns) 25. The cytotoxicity of **Lipi-QA** was also evaluated by MTT assay (**Figure S13**). Incubation of HeLa cells with a probe concentration of 5 μM for 24 h, the cell viability remained at a high level. In general, staining cells with a probe concentration of 0.1‒2 μM for 2 h (**Figure S6** and **S7**) could provide sufficient fluorescence brightness for confocal imaging and STED super-resolution imaging.

### Time-Lapse Three-Dimensional Confocal Imaging with Lipi-QA

**Figure 2:**
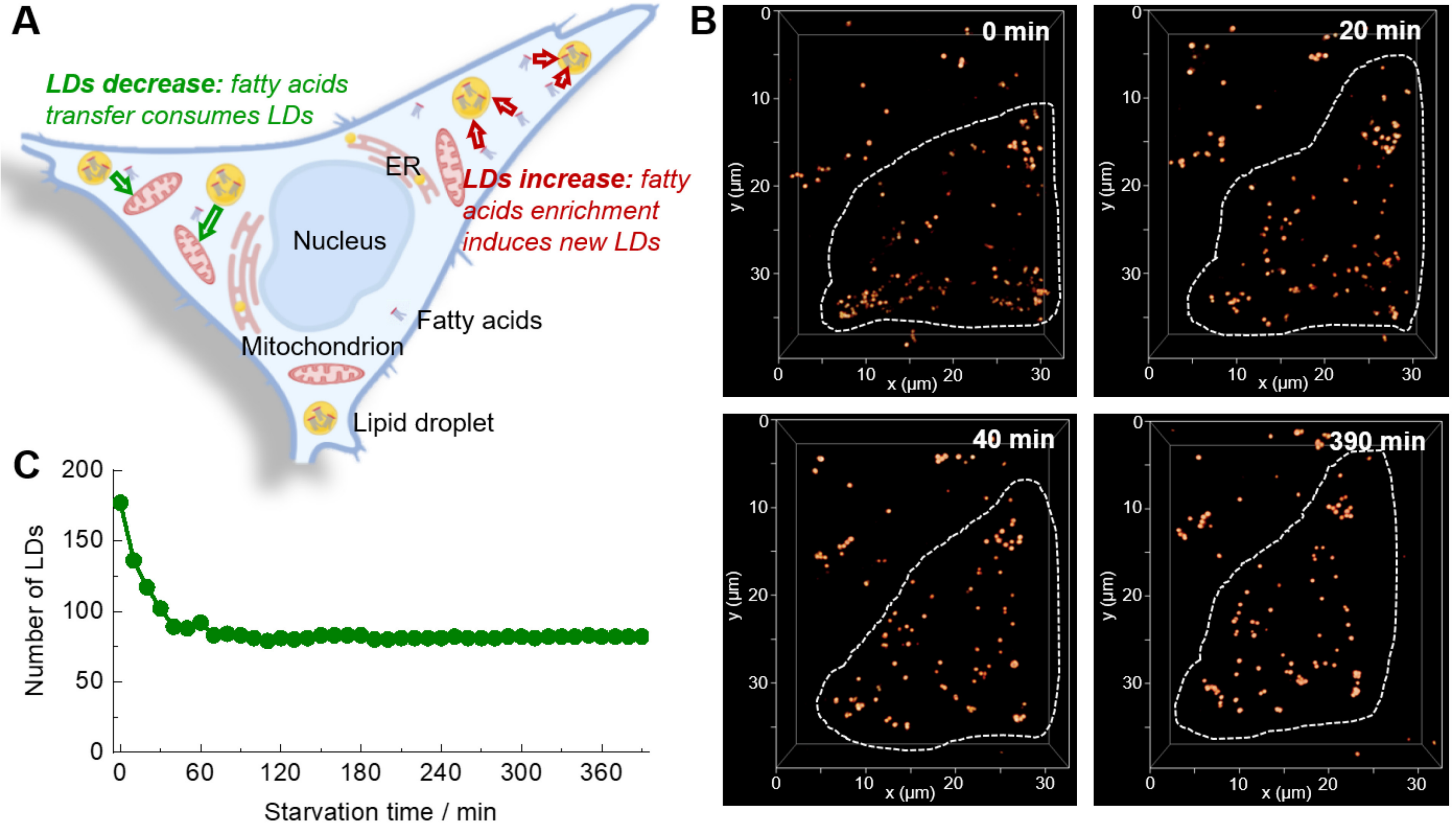
(A) A controversial question that the number of LDs would increase or decrease upon starvation. (B) Time-lapse 3D confocal imaging of living HeLa cells labelled with **Lipi-QA** (2 μM, 2 h) under starvation stimulation. The 3D images (*xyz*: 32 × 40 × 14 μm^3^; top view) at different time spots are shown. *λ*_ex_ = 488 nm, *λ*_em_ = 500‒700 nm. (C) The change of LDs number of the HeLa cell upon starvation.

The 3D confocal imaging is a powerful tool to visualize the spatial distribution of cellular organelles and count their numbers or amounts. Further adding the temporal dimensionality (time-lapse) to 3D confocal imaging, *i.e.* the 4D confocal imaging, would be even able to in-situ monitor the changing process in 3D space. Absolutely, the 4D confocal imaging is highly attractive for biological study, but at the same time is very challenging because of the serious photobleaching of fluorescent probe. Moreover, the staining selectivity of probe also plays a critical role in the visualization of spatial distribution of LDs (**Figure S14**). The super-photostability and high LDs selectivity of** Lipi-QA** inspired us to conduct the 4D confocal imaging. In consideration of that the study of starvation-induced change of LDs has attracted much attention for cell biologists and there is an important controversial question that the number of LDs would increase or decrease upon starvation (**Figure 2A**) 26-29, we herein performed the 4D confocal imaging with **Lipi-QA** to in-situ track the change of LDs. For this experiment, the live HeLa cells pre-stained with **Lipi-QA** were kept in HBSS medium for starvation and imaging. The 3D confocal image was recorded with a precise imaging condition: a high pixel resolution of 45.1 nm × 45.1 nm for *xy* frame and a small step of 300 nm for *z*-axis. Based on the 48 confocal frames in a *z*-depth of 14.1 μm, one 3D confocal image could be reconstructed with high quality. In a long period of 6.5 h, the 3D images were repeatedly accumulated up to 40 times with a time interval of 10 min, thus providing the 4D confocal imaging (**Figure 2B** and **Figure S15**). These was a very impressive imaging result since the 4D imaging consisted of about 2000 confocal images. Moreover, almost no photobleaching was observed during repeatedly imaging. Based on the 4D imaging result, it was able to directly counter the number of LDs at different time (**Figure 2C**). We thus could certainly state that the number of LDs significantly decreased rather than increased upon starvation. However, the exact reason is not clear to us, we will further investigate the cause of this phenomenon in a follow-up study. Moreover, the 4D confocal imaging could even give the information regarding decrease speed of LDs upon starvation: decreased very fast in the beginning 40 min, and almost didn't change after that.

### STED Super-Resolution Imaging with Lipi-QA

The characteristic features of **Lipi-QA**, including the super-photostability, high LDs selectivity, and long fluorescence lifetime, suggested its promising utility for STED super-resolution imaging. The STED super-resolution microscope employs an additional donut depletion laser based on the confocal microscope. The fluorescent molecules in the outer ring of light spot within the donut depletion laser enveloped area will be erased by stimulated radiation when the fluorescent molecules are excited from the ground state to the excited state, thus enabling super-resolution imaging. Higher resolution is achieved as the hollow zone of donut depletion laser shrinks as the STED depletion laser intensity increases. Therefore, compared to confocal imaging, STED super-resolution imaging enable to break the resolution limit of light diffraction and visualize the nanoscale dynamic process of LDs in living cells with high spatial and temporal resolution. In other word, STED super-resolution imaging would provide much more clear LDs than confocal imaging. The depletion efficiency of **Lipi-QA** under the continuous wave STED laser (660 nm) was then studied. The 592 nm STED laser wasn't employed because this laser would directly excite **Lipi-QA**. The living HeLa cells pre-stained with this probe was imaged under the same excitation power (*λ*_ex_ = 488 nm) while gradually increasing the STED laser intensity (**Figure S16**). Consequently, the fluorescence signals of images were significantly quenched by the STED laser. After plotting the fluorescence intensity of each image as a function of the STED laser intensity, the saturation intensity (*I*_sat_) of **Lipi-QA** was determined to be 1.6 MW cm^-2^. This *I*_sat_ value is dramatically lower than that of the gold standard STED super-resolution imaging fluorescent probe ATTO 647N (10‒20 MW cm^-2^) 30-32, revealing the high depletion efficiency of **Lipi-QA**. This result should be directly relative to the long fluorescence lifetime of **Lipi-QA**, because the *I*_sat_ is inversely proportional to the lifetime.

**Figure 3:**
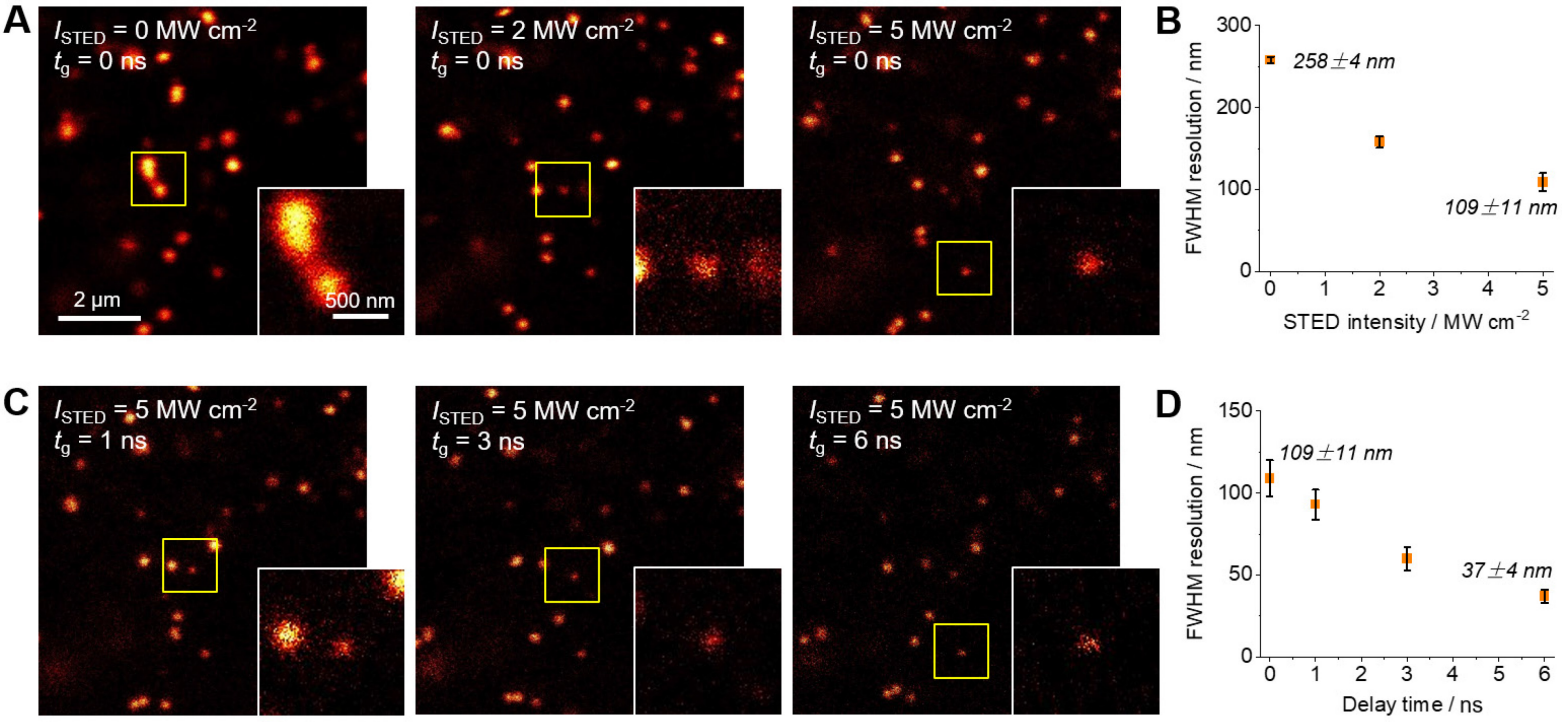
(A‒B) The STED laser intensity-dependent resolution and (C‒D) the gating detection delay time-dependent resolution. The STED super-resolution images of living HeLa cells stained with **Lipi-QA** (2 μM, 2 h) were recorded under the excitation of 488 nm, the depletion of 660 nm (STED laser intensity *I*_STED_ of 0, 2, and 5 MW cm^-2^), and the gating detection (delay time *t*_g_ of 0, 1, 3, and 6 ns). Scale bar: 2 μm. The enlarged views of the regions marked with squares are shown in the insets. Scale bar: 500 nm.

The STED laser intensity-dependent resolution of **Lipi-QA** was then studied. The living HeLa cells labelled with this probe was imaged under the excitation of 488 nm and the depletion of 660 nm. Upon increasing the STED laser intensity, the images of LDs become sharper and sharper (**Figure 3A**). Accordingly, the full width at half maxima (FWHM) resolution of LDs are significantly improved from 258 ± 4 nm under a STED intensity of 0 MW cm^-2^ (*i.e.* confocal image) to 158 ± 7 nm under a STED intensity of 2 MW cm^-2^, and further to 109 ± 11 nm under a STED intensity of 5 MW cm^-2^ (**Figure 3B** and **Figure S17A**). Moreover, the long fluorescence lifetime character of **Lipi-QA** enabled to apply time-gated detection to further improve the resolution of STED images (**Figure 3C**) 19. Under the STED laser intensity of 5 MW cm^-2^, the STED imaging with gate delay times (*t*_g_) of 1 ns, 3 ns or 6 ns provided FWHM resolutions of 93 ± 9 nm, 60 ± 7 nm, or even up to 37 ± 4 nm (**Figure 3D** and **Figure S17B**). The resolution of 37 ± 4 nm is substantially broken the diffraction limit of light (~250 nm) and represents the highest resolution of fluorescence imaging of LDs up to date (**Table S3**) 11-16. This is also a state-of-the-art resolution of living cell STED super-resolution imaging (**Table S4**) 33-45. This high resolution should be closely related to the long fluorescence lifetime which not only decreases the *I*_sat_ intensity of **Lipi-QA** (**Supplementary Information**, Discussion of the resolution) but also makes it to be capable of time-gated detection 19.

More importantly, the super-photostability of **Lipi-QA** enabled to even perform time-lapse STED super-resolution imaging at the nanoscale. Under the strong STED laser intensity of 5 MW cm^-2^ and time-gated detection (*t*_g_ = 6 ns), the dynamics of LDs in living HeLa cells were successfully tracked up to 500 STED frames (10.9 min, 0.763 fps) while maintaining meaningful fluorescence signals (**Movie S1**). This is very impressive, because the intense STED laser generally causes fast photobleaching of fluorescent probes. Even the recently developed photostable ones still encounter the photobleaching problem under STED imaging 33-48. Time-lapse STED imaging of **Lipi-QA** with hundreds of frames and high resolution is much appreciated for the biological study of LDs in living cells at the unprecedented nanoscale resolution.

**Figure 4:**
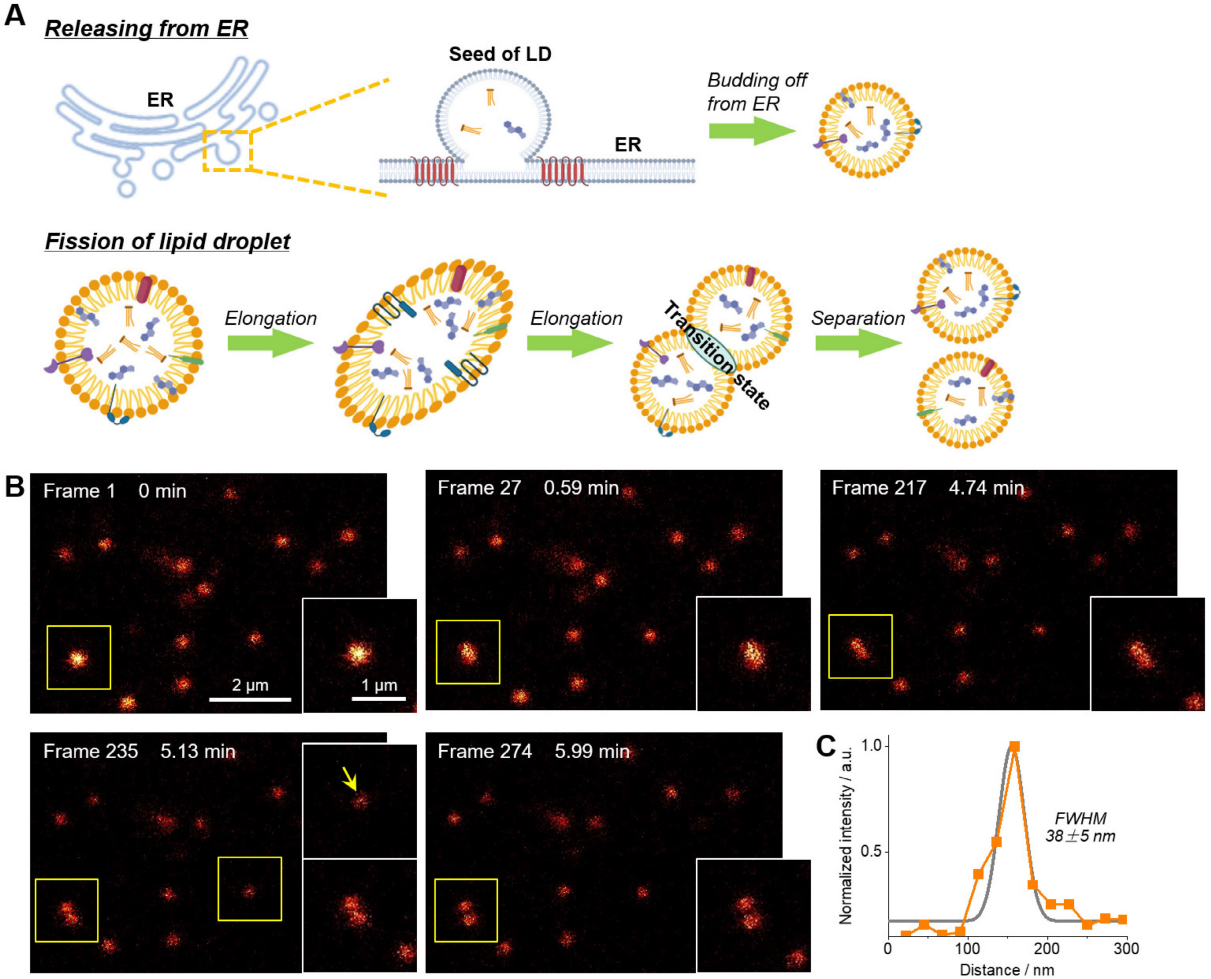
(A) Schematic diagram of two ways of LDs formation. (B) Time-lapse STED super-resolution imaging with **Lipi-QA** (2 μM, 2 h) to reveal the fission process of LD at nanoscale resolution. Scale bar of 2 μm for the images and 1 μm for the enlarged views. (C) The signal intensity profile crossed the LD (indicated by the arrow), the FWHM resolution was determined from Gaussian fitting (gray line). All the STED images are shown in raw data without deconvolution or photobleaching correction.

The fission of LD has been proposed to be one of two ways to form new LDs (**Figure 4A**) 49-51. However, much more experimental evidences, especially the in-situ visualization of LD fission, are absolutely required to support this speculation. In this context, time-lapse STED imaging were employed to track the fission process of LD at the nanoscale (**Figure 4B‒C** and **Movie S2**). Within 6 min (274 frames), a spherical LD gradually elongated and then divided into two smaller LDs with diameters of around 150 nm. It should be noted that such fission process cannot be visualized via confocal imaging due to the insufficient resolution (∼250 nm). The electron microscopy with high resolution is also not capable for this purpose because only fixed cells can be imaged. To the best of our knowledge, this is the first report of visualization of LD fission with nanoscale resolution.

### Study the Change of LDs under Starvation Condition by STED Super-resolution Imaging

**Figure 5:**
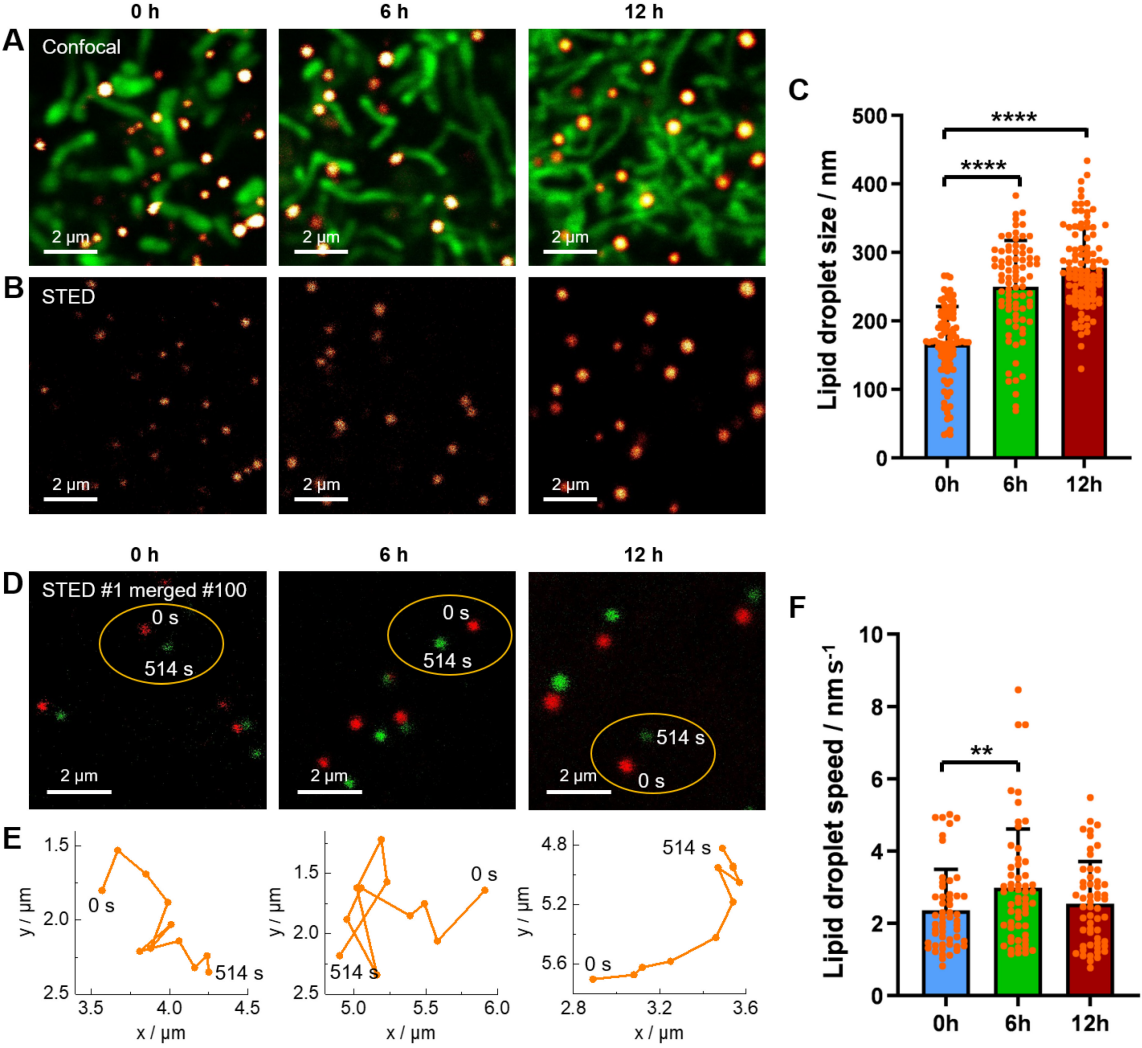
(A‒B) Confocal images and STED super-resolution images of living HeLa cells upon starvation for different time (0 h, 6 h, and 12 h). The LDs (red hot color) and mitochondria (green color) were labelled with** Lipi-QA** (2 μM, 2 h) and TMRM (200 nM, 0.5 h), respectively. Scale bar: 2 μm. (C) The size distribution of LDs under starvation condition. (D) The merged images of the 1st (0 s) and 100th (514 s) frames of time-lapse STED super-resolution imaging of living HeLa cells under starvation condition. Scale bar: 2 μm. (E) The motion paths of LDs within 514 s. (F) The speed distribution of LDs under starvation condition.

In the before-mentioned part, we have revealed the starvation-induced decrease of LDs in number by 4D confocal imaging. Here, we would like to further demonstrate the starvation-induced change of LDs in size and in speed of movement by STED super-resolution imaging. The living HeLa cells pre-stained with **Lipi-QA** and tetramethylrhodamine methyl ester (TMRM, mitochondria probe) were kept in HBSS medium for starvation of 0 h, 6 h and 12 h. The starvation condition of cells could be confirmed by the morphology change of mitochondria: the mitochondria displayed short rods structures at 0 h, elongated strips structures at 6 h, and interwoven into networks at 12 h (**Figure 5A**) 52. Under the confocal imaging, it seems that the sizes of LDs (around 250‒500 nm) are similar with each other, without significant change upon starvation. However, in consideration of the resolution limitation, the confocal imaging may be not able to reflect the true result of LDs sizes. In other word, a LD with actual size of 100 nm would be visualized to be about 300 nm by confocal imaging. In this context, the STED super-resolution imaging would be highly appreciated to precisely determine the LDs sizes. Indeed, many LDs with sizes of less than 200 nm or even 100 nm have been successfully visualized by STED imaging (**Figure 5B**). After recording many STED images, it has been provided a statistical result of LDs sizes: 166 ± 55 nm (*n* = 98), 250 ± 68 nm (*n* = 83), and 277 ± 59 nm (*n* = 101) for starvation of 0 h, 6 h and 12 h, respectively (**Figure 5C**). The LDs sizes are gradually increased upon starvation of 12 h. This result may be related to that the autophagy processes of LDs and other cellular organelles would generate the fatty acids which enter and enlarge the residual LDs 28,29,53.

Besides of precise determination of the LDs sizes, time-lapse STED live imaging enabled to further track the dynamics of LDs at nanoscale and thus to calculate the LDs speeds. In this experiment, the living HeLa cells pre-stained with **Lipi-QA** were starved in HBSS medium for various time (0 h, 6 h and 12 h) and the time-lapse STED imaging consisted of 100 frames (514 s) were recorded (**Figure 5D**). Further extending time, some LDs may move out from the imaging view. During the STED imaging, the positions of LDs were marked with a time interval of 51.4 s (10 STED frames), thus providing the motion paths of LDs (**Figure 5E**). Although further shortening the time interval was possible, the distances of LDs movements would be too small and the measurement errors of distances may be increased. Based on the motion paths of LDs, the average values of LDs speeds could be calculated. After recording many time-lapse STED imaging experiments, we have got a statistical result of LDs speeds: 2.36 ± 1.14 nm s^-1^ (*n* = 53), 2.99 ± 1.61 nm s^-1^ (*n* = 58) and 2.55 ± 1.17 nm s^-1^ (*n* = 55) for starvation of 0 h, 6 h and 12 h, respectively (**Figure 5F**). In comparison to the normal conditions (starvation of 0 h), the LDs speeds are increased upon starvation of 6 h. This result may be due to that the fatty acids metabolism would be the dominated process to provide energy for cells under starvation and consequently the activity of LDs should be enhanced to transport the fatty acids 28. While further starvation of 12 h, we speculate that the decrease of LDs speeds may be related to the damage of cells. We will continue to study this issue through relevant experiments in the future.

## Discussion

We have developed a superior organic molecular probe **Lipi-QA** for LDs fluorescence imaging and biological study. This fluorescent probe was designed to overcome the three principal drawbacks of conventional LDs probes: photostability, LDs specificity and fluorescence lifetime. Firstly, the insufficient photostability of conventional LDs probes (*i.e.* rapid photobleaching) would directly hamper the repeated acquisition of frames for time-lapse imaging. In this point of view, it was impressive to conduct the time-lapse 3D confocal imaging (about 2000 confocal frames) and the time-lapse STED super-resolution imaging (up to 500 STED frames). Secondly, the high LDs specificity of probe **Lipi-QA** is important for the clear visualization of LDs with high contrast. The non-specific staining of conventional LDs probes toward plasma membrane and cytoplasm would cause strong fluorescence background, especially in the 3D imaging. Thirdly, the long fluorescence lifetime of probe **Lipi-QA** is critical for the STED super-resolution imaging with a state-of-the-art resolution (37 nm). On one hand, the long fluorescence lifetime largely contributed to the low saturation intensity, thus improving the resolution. On the other hand, the long fluorescence lifetime enabled to apply time-gated detection to remove short lived fluorescence signal, hence to further improve the resolution.

Taking advantages of the before-mentioned features, we could conduct many interesting but seldom ever realized LDs imaging experiments, and thus get new insights into the biological study of LDs. For example, we have successfully performed the long-term time-lapse 3D confocal imaging to in-situ monitor the changing process of LDs under starvation stimulation. Based on the imaging result, we have surely answered an important and controversial question that the number of LDs would significantly decrease rather than increase upon starvation. We have successfully accomplished the time-lapse STED super-resolution imaging to track the dynamics of LDs at nanoscale resolution. Accordingly, the fission process of LD has been impressively visualized at nanoscale for the first time. We have further employed STED super-resolution imaging to study the change of LDs in size and in speed of movement under starvation stimulation. Undoubtedly, the high resolution of STED imaging with **Lipi-QA** was essential to precisely determine the LDs sizes and speeds at nanoscale. Further revealing the new biological functions of LDs by using this excellent fluorescent probe as well as developing a series of quinacridone-based photostable fluorescent probes for other organelles (such as cytomembrane, mitochondrion, lysosome and so on) is ongoing by our group and collaborators. In addition, we would be very happy to freely share this fluorescent probe for cell biologist community.

## Materials and methods

### Materials

Chemical synthesis protocols and full characterization data of the fluorescent probe **Lipi-QA** and its analogies **1**‒**2** can be found in the Supplementary information.

### General staining procedure of fluorescent probes

Live cells (HeLa, HepG2, HT22 and U251) were stained in DMEM+ containing 2 μM fluorescent probe (**Lipi-QA**, molecules **1** or **2**, Nile Red, or BODIPY 493/503) and 1% DMSO for 2 h in a CO_2_ incubator. Then, the cells were washed three times with fresh medium to remove the free probes, and kept in HBSS for confocal imaging and STED imaging.

### Time-lapse three-dimensional confocal imaging

The imaging experiments were conducted with *xyzt* shooting mode under Leica TCS SP8 imaging system with following set: *λ*_ex_ = 488 nm, *λ*_em_ = 500‒640 nm, a scan speed of 100 Hz, a pixel resolution of 45.1 nm × 45.1 nm, a *z*-step of 300 nm, a total of 40 sets of 3D imaging, about 2000 confocal images were taken. In addition, a microscopy-suited incubator was employed to control the temperature (37 °C) and CO_2_ concentration (5%).

### STED imaging

The Leica TCS SP8 STED system equipped with two continuous wave depletion lasers (CW-STED: 592 nm or 660 nm) was used for STED imaging. A HyD detector and a STED WHITE objective (100x/1.40 OIL) were employed. Unless otherwise noted, the STED images were acquired with excitation at 488 nm (WLL), emission in the range of 500‒640 nm, time-gated detection range of 6‒12 ns, and depletion at 660 nm (CW-STED, 5 MW cm^-2^). In general, the images were recorded with a pixel resolution of 16.2 nm × 16.2 nm, a scan speed of 100 Hz, and a bidirectional model. The images were processed using ImageJ. The full width at half maximum (FWHM) resolution was determined based on the Gaussian fitting of the signal intensity profiles crossed the LDs.

## Supplementary Material

Supplementary methods, figures, tables, discussion, movie legends, and NMR spectra.

Supplementary movie 1.

Supplementary movie 2.

Supplementary crystal structure of Lipi-QA.

## Abbreviations

LDs: lipid droplets
STED: stimulated emission depletion
ER: endoplasmic reticulum
PALM: photoactivated localization microscopy
TD-DFT: time dependent density functional theory
