# Supplementary material for: A new organic molecular probe as a powerful tool for fluorescence imaging and biological study of lipid droplets
Source: Theranostics. 2023 Jan 1;13(1):95–105. doi: 10.7150/thno.79052 (PMC9800742; doi:10.7150/thno.79052)
Supplement: Supplementary file 1 — Supplementary methods, figures, tables, discussion, movie legends, and NMR spectra. [file thnov13p0095s1.pdf]

Supplementary Information for

# A New Organic Molecular Probe as a Powerful Tool for Fluorescence Imaging and Biological Study of Lipid Droplets

Ri Zhou[1], Chenguang Wang*[1], Xishuang Liang[1], Fangmeng Liu[1], Peng Sun[1], Xu Yan[1], Xiaoteng Jia[1], Xiaomin Liu[1], Yue Wang[2], and Geyu Lu*[1,3]

1. State Key Laboratory of Integrated Optoelectronics, Key Laboratory of Advanced Gas Sensors of Jilin Province, College of Electronic Science & Engineering, Jilin University, 2699 Qianjin Street, Changchun 130012, China
2. State Key Laboratory of Supramolecular Structure and Materials, College of Chemistry, Jilin University, 2699 Qianjin Street, Changchun 130012, China
3. International Center of Future Science, Jilin University, 2699 Qianjin Street, Changchun 130012, China

Corresponding authors Email: wangchenguang@jlu.edu.cn; lugy@jlu.edu.cn

## This PDF file includes:

1. Supplementary methods
2. Supplementary chemical synthesis
3. **Tables S1** to **S4**
4. **Figure S1** to **S17**
5. Discussion of the resolution
6. Captions for **Movies S1** to **S2**
7. NMR spectra of synthesized compounds

## Other Supplementary materials for this manuscript include the following:

CIF crystal structure file of **Lipi-QA**

**Movies S1** to **S2**

## 1. Supplementary methods

**Single Crystal X-ray Measurements.** Single crystal X-ray diffraction data were collected on a Rigaku RAXIS-PRID diffractometer using the $\omega$-scan mode with graphite monochromator Mo • K $\alpha$ radiation. The structures were solved with direct methods using the SHELXTL programs and refined with full-matrix least-squares on $F^2$. Non-hydrogen atoms were refined anisotropically. The positions of hydrogen atoms were calculated and refined isotropically. The crystals of Lipi-QA were prepared by slow evaporation of its solution with mixture solvents of $CH_2Cl_2$ and petroleum ether at room temperature. The crystal structure of fluorescent probe Lipi-QA has been deposited with the Cambridge Crystallographic Data Centre (CCDC: 2154920).

**UV-vis and fluorescence spectroscopy.** UV-vis absorption spectra of solutions were measured with a Shimadzu UV-2550 spectrometer. Emission spectra of solutions were measured with an Ocean QE Pro fibre optic spectrometer. Absolute fluorescence quantum yields ($\Phi_F$) of solutions were determined with an Ocean QE Pro fibre optic spectrometer equipped with a calibrated integrating sphere system. Fluorescence lifetimes of solutions were measured using an Edinburgh FLS920 spectrometer equipped with a 365 nm LED excitation source. The fluorescence decay profiles of solutions were fitted reasonably well with a single exponential function.

**Cell culture.** In general, HeLa, HepG2, HT22 and U251 cells were cultured in Dulbecco's modified Eagle's medium (DMEM, high glucose, pyruvate) containing 10% fetal bovine serum (FBS) and 1% Antibiotic-Antimycotic (AA) at 37 °C in a humidified 5% (vol/vol) $CO_2$ incubator.

**Cell viability assays.** The effect of probe Lipi-QA on cell viability was analyzed using 3-(4,5-dimethylthiazol-2-yl)-2,5-diphenyltetrazolium bromide (MTT). Cells were seeded into a flat-bottomed 96-well plate ($1 \times 10^4$ cells/well) and incubated in DMEM containing 10% FBS (DMEM+) at 37 °C in a 5% $CO_2$/95% air incubator for 24 h. The medium was then replaced with a culture medium DMEM+ containing various concentrations of Lipi-QA (0, 1.0, 2.0, 5.0, 10.0 and 20.0 μM) and 1% DMSO. After incubation for 24 h, MTT reagent (final concentration, 0.5 mg/mL) was added to each well, and the plates were incubated for another 4 h in a $CO_2$ incubator. Excess MTT tetrazolium solution was then removed. After the formazan crystals were solubilized in DMSO (100 μL/well) for 30 min at room temperature, the absorbance of each well was measured by a microplate reader (Bio-Tek Instruments, Inc) with an excitation at 630 nm.

**Co-staining experiments.** Cells were seeded in glass-bottom dishes two days before imaging. After removal of the medium, HeLa cells were stained in DMEM+ containing probes (2 μM Lipi-

QA and 1 μM Ph-Red, or 2 μM molecule **1** and 1 μM Ph-Red, or 2 μM molecule **2** and 500 nM Ph-Red) and 1% DMSO for 2 h in a $CO_2$ incubator. HepG2, HT22 and U251 cells were stained in DMEM+ containing probes (2 μM Lipi-QA and 1 μM Ph-Red) and 1% DMSO for 2 h in a $CO_2$ incubator. After washing with fresh medium to remove the free probes, the cells were kept in HBSS for imaging. The confocal images were recorded using a Leica TCS SP8 microscope with following set: $\lambda_{ex}$ = 488 nm, $\lambda_{em}$ = 500–540 nm for Lipi-QA and molecules **1**–**2**; $\lambda_{ex}$ = 488 nm, $\lambda_{em}$ = 680–720 nm for Rh-Red.

**General staining procedure of fluorescent probes.** Live cells (HeLa, HepG2, HT22 and U251) were stained in DMEM+ containing 2 μM fluorescent probe (Lipi-QA, molecules **1** or **2**, Nile Red, or BODIPY 493/503) and 1% DMSO for 2 h in a $CO_2$ incubator. Then, the cells were washed three times with fresh medium to remove the free probes, and kept in HBSS for confocal imaging and STED imaging.

**Confocal imaging.** For the comparison of LDs staining specificity of fluorescent probes in different cells, the confocal images were recorded using a Leica TCS SP8 microscope with following set: $\lambda_{ex}$ = 488 nm, $\lambda_{em}$ = 500–700 nm. For the comparison of the photostability of fluorescent probes in HeLa cells, the confocal images were recorded using a Nikon A1RMP microscope with an identical power (150 mW, output power of 10%) of excitation laser (488 nm). And the staining concentration of the three probes was 2 μM. The total signal intensities of each image were measured with ImageJ, normalized to the value of the first image and plotted as a function of the number of recorded confocal images. Unless otherwise stated, the confocal images of cellular LDs stained with Lipi-QA, Nile Red and BODIPY were recorded with the following set: $\lambda_{ex}$ = 488 nm, $\lambda_{em}$ = 500–700 nm.

**Multicolor confocal imaging.** Live HeLa cells were stained in DMEM+ containing Lipi-QA (2 μM) and 1% DMSO for 2 h in a $CO_2$ incubator. After washing with fresh medium, the cells were then stained in DMEM+ containing MitoTracker Deep Red (50 nM), Hoechst 33342 (20 μM) and 1% DMSO for 30 min. After washing, the cells were further stained in DMEM+ containing LysoTracker Red (200 nM) and 1% DMSO for 30 min. Without washing, the cells were imaged by a Leica TCS SP8 microscope with following set: $\lambda_{ex}$ = 405 nm, $\lambda_{em}$ = 415–465 nm for Hoechst 33342; $\lambda_{ex}$ = 488 nm, $\lambda_{em}$ = 500–560 nm for Lipi-QA; $\lambda_{ex}$ = 570 nm, $\lambda_{em}$ = 580–615 nm for LysoTracker Red; $\lambda_{ex}$ = 650 nm, $\lambda_{em}$ = 660–750 nm for MitoTracker Deep Red. The four channels were detected via a line-by-line sequential scanning.

**Determination of fluorescence lifetime of Lipi-QA in lipid droplets.** Time-resolved confocal fluorescence imaging of HeLa cells stained with Lipi-QA was performed with a Leica TCS SP8 system. The images were recorded by sequentially changing the delay time ($t_g$ = 0–8.5 ns, 0.5 ns step) with a constant gate width of the detector ($\Delta t$ = 3.5 ns). The total fluorescence signal intensity of each image was plotted as a function of the delay time. The decay curve was fitted by a single exponential function to calculate the fluorescence lifetime ($\tau$) of the probe in lipid droplets.

**Time-lapse three-dimensional confocal imaging.** The imaging experiments were conducted with *xyzt* shooting mode under Leica TCS SP8 imaging system with following set: $\lambda_{ex}$ = 488 nm, $\lambda_{em}$ = 500–640 nm, a scan speed of 100 Hz, a pixel resolution of 45.1 nm × 45.1 nm, a *z*-step of 300 nm, a total of 40 sets of 3D imaging, about 2000 confocal images were taken. In addition, a microscopy-suited incubator was employed to control the temperature (37 °C) and $CO_2$ concentration (5%). The 3D confocal images were reconstructed through the LAS X software equipped by the commercial Leica TCS SP8 microscopy. Based on the maximum intensity projection conducted by the ImageJ software, LDs in the 3D confocal images could be displayed in the 2D images, thus the number of LDs could be counted.

**STED imaging.** The Leica TCS SP8 STED system equipped with two continuous wave depletion lasers (CW-STED: 592 nm or 660 nm) was used for STED imaging. A HyD detector and a STED WHITE objective (100x/1.40 OIL) were employed. Unless otherwise noted, the STED images were acquired with excitation at 488 nm (WLL), emission in the range of 500–640 nm, time-gated detection range of 6–12 ns, and depletion at 660 nm (CW-STED, 5 MW $cm^{-2}$). In general, the images were recorded with a pixel resolution of 16.2 nm × 16.2 nm, a scan speed of 100 Hz, and a bidirectional model. The images were processed using ImageJ. The full width at half maximum (FWHM) resolution was determined based on the Gaussian fitting of the signal intensity profiles crossed the LDs.

**Two-color confocal imaging.** Live HeLa cells were stained in DMEM+ containing 2 μM Lipi-QA and 1% DMSO for 2 h in a $CO_2$ incubator. After washing with fresh medium, the cells were further stained in DMEM+ containing 50 nM tetramethylrhodamine methyl ester (TMRM) and 1% DMSO for 20 min in a $CO_2$ incubator. Then, the cells were washed three times with fresh medium to remove the free probes, and kept in HBSS for confocal imaging. Lipi-QA and TMRM

were excited at 488 and 560 nm and detected for 500–550 nm ($t_g$ = 6–12 ns) and 570–640 nm ($t_g$ = 0–12 ns), respectively, in a line-by-line sequential scan mode.

**Statistical analysis.** All data were expressed as mean ± S.D., and differences between groups were analyzed by one-way analysis of variance (ANOVA). Tukey's post hoc test was performed by data statistical analysis software (SPSS Statistics, Version number: 18.0.0.282). $p < 0.05$ was considered as statistically significant, expressing as one star, $p < 0.01$ as two stars, and $p < 0.001$ as three stars.

## 2. Supplementary chemical synthesis

**General.** $^{1}H$ and $^{13}C$ NMR spectra were recorded with a Zhongke-Niujin AS 400 spectrometer (400 MHz for $^{1}H$ and 101 MHz for $^{13}C$) in $CDCl_3$ or DMSO-$d_6$. The chemical shifts in $^{1}H$ NMR and $^{13}C$ NMR spectra were reported in $\delta$ ppm using tetramethylsilane as an internal standard. Mass spectra was recorded on a Thermo Fisher ITQ1100 GC/MS mass spectrometer. All reactions were performed under a $N_2$ atmosphere, unless otherwise stated. Commercially available solvents and reagents were used without further purification unless otherwise mentioned.

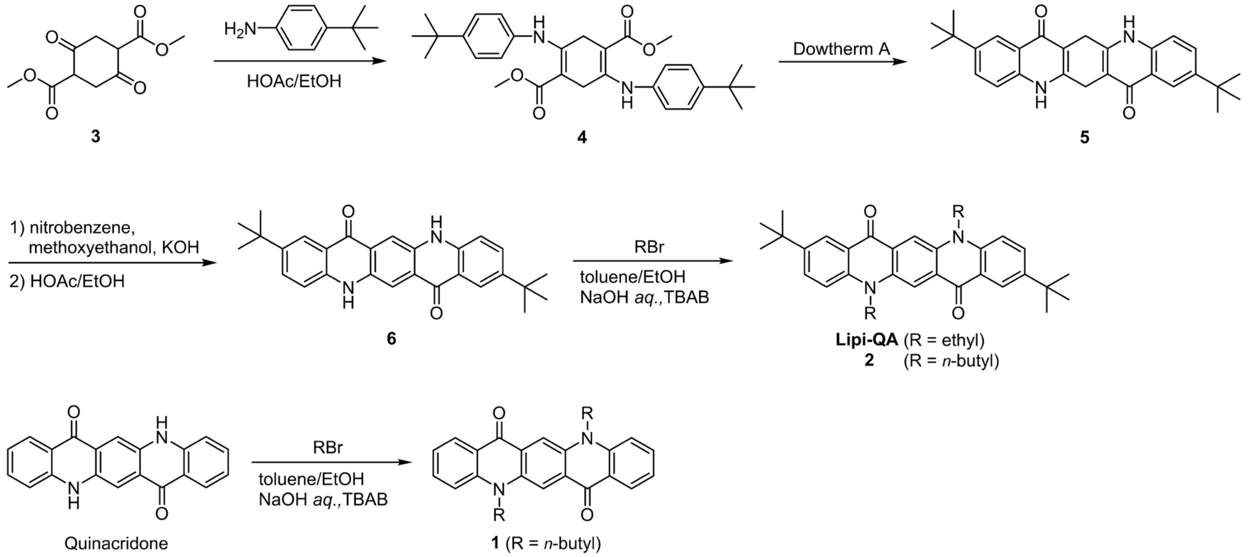


**Compound 4.** To a solution of compound **3** (11.4 g, 50.0 mmol) in ethanol (100 mL) and acetic acid (50 mL) was added *tert*-butylaniline (20.0 mL, 125 mmol), and then the mixture was refluxed for 12 h. After cooling to room temperature, the mixture was filtrated. The precipitated solids were washed with methanol for three times and dried to obtain 23.5 g (48.0 mmol, 96%) compound **4** as pale-yellow powders. $^{1}H$ NMR (400 MHz, $CDCl_3$): $\delta$ 7.40 (d, $J$ = 8.3 Hz, 4H), 7.08 (d, $J$ = 8.3 Hz, 4H), 3.70 (s, 6H), 3.43 (s, 4H), 1.38 (s, 18H).

**Compound 5.** A solution of compound **4** (21.0 g, 42.9 mmol) in Dowtherm A (200 mL) was refluxed for 12 h. After cooling to room temperature, the mixture was filtrated. The precipitated solids were washed with methanol for three times and dried to obtain 9.50 g (22.3 mmol, 52%) compound **5** as pale pink powders. ESI-MS: 424.20 $[M\text{–}H]^+$ (calcd: 425.23).

**Compound 6.** To a solution of compound **5** (8.00 g, 18.8 mmol) in nitrobenzene (50 mL) and ethylene glycol unimethyl ether (100 mL) was added KOH (8.00 g, 143 mmol), and then the mixture was refluxed for 4 h. After removing most of the solvents under reduced pressure, ethanol

(100 mL) and acetic acid (100 mL) were successively added to the reaction system and the resulted mixture was further refluxed for 3 h under air condition. After cooling to room temperature, the mixture was filtrated. The precipitated solids were washed with methanol for three times and dried to obtain 7.97 g (18.8 mmol, ~100%) compound **6** as dark red powders. $^1$H NMR (400 MHz, DMSO-$d_6$): $\delta$ 8.50 (s, 2H), 8.21 (d, $J$ = 2.1 Hz, 2H), 7.90 (dd, $J$ = 8.7, 1.8 Hz, 2H), 7.51 (d, $J$ = 8.8 Hz, 2H), 1.39 (s, 18H). ESI-MS: 424.15 $[M–H]^+$ (calcd: 423.22).

**Lipi-QA.** To a mixture of compound **6** (1.00 g, 2.36 mmol) and tetrabutylammonium bromide (TBAB, 120 mg, 0.372 mmol) in toluene (20 mL) and ethanol (2 mL) was added sodium hydroxide aqueous solution (25.0 mM, 20 mL) and bromoethane (3.7 mL, 50 mmol) successively, and the resulting mixture was refluxed for 48 h. After cooling to room temperature, the mixture was diluted with water (200 mL) followed by extraction with dichloromethane (300 mL). The organic layer was dried over anhydrous magnesium sulfate and filtered. After concentration of the filtrate under reduced pressure, the resulting mixture was purified by silica gel column chromatography (methylene chloride/ethyl acetate = 20/1). Further purification via recrystallization from methylene chloride/methanol provided 650 mg (1.35 mmol, 57%) Lipi-QA as orange-red powders. $^1$H NMR (400 MHz, $CDCl_3$): $\delta$ 9.00 (s, 2H), 8.64 (d, $J$ = 2.4 Hz, 2H), 7.91 (dd, $J$ = 9.1, 2.5 Hz, 2H), 7.59 (d, $J$ = 9.2 Hz, 2H), 4.73 (q, $J$ = 7.3 Hz, 4H), 1.66 (t, $J$ = 7.2 Hz, 6H), 1.45 (s, 18H). $^{13}$C NMR (101 MHz, $CDCl_3$): $\delta$ 178.21, 143.72, 140.06, 135.15, 132.81, 126.20, 123.55, 120.62, 114.29, 113.10, 41.04, 34.48, 31.33, 12.42. The structure of Lipi-QA was further confirmed by single crystal X-ray diffraction analysis.

**Compound 2.** This compound was prepared in a similar manner as described for Lipi-QA using compound **6** and *n*-bromobutane as starting materials. $^1$H NMR (400 MHz, $CDCl_3$): $\delta$ 8.83 (s, 2H), 8.60 (d, $J$ = 2.4 Hz, 2H), 7.88 (dd, $J$ = 9.1, 2.5 Hz, 2H), 7.49 (d, $J$ = 9.1 Hz, 2H), 4.60–4.49 (m, 4H), 2.05–1.94 (m, 4H), 1.72–1.62 (m, 4H), 1.44 (s, 18H), 1.12 (t, $J$ = 7.4 Hz, 6H). $^{13}$C NMR (101 MHz, $CDCl_3$): $\delta$ 178.13, 143.78, 140.39, 135.47, 132.86, 126.06, 123.51, 120.55, 114.53, 113.34, 46.05, 34.49, 31.32, 29.18, 20.28, 13.92.

**Compound 1.** This compound was prepared in a similar manner as described for Lipi-QA using quinacridone and *n*-bromobutane as starting materials. $^1$H NMR (400 MHz, $CDCl_3$): $\delta$ 8.71 (s, 2H), 8.56 (dd, $J$ = 8.0, 1.3 Hz, 2H), 7.78–7.70 (m, 2H), 7.47 (d, $J$ = 8.8 Hz, 2H), 7.22 (t, $J$ = 7.5 Hz, 2H), 4.55–4.44 (m, 4H), 2.07–1.95 (m, 4H), 1.72–1.60 (m, 4H), 1.11 (t, $J$ = 7.4 Hz, 6H). $^{13}$C

NMR (101 MHz, $CDCl_3$): $\delta$ 178.03, 142.20, 135.64, 134.58, 128.01, 126.17, 121.00, 120.82, 114.59, 113.46, 46.12, 29.12, 20.26, 13.92.

## 3. Supplementary Tables S1 to S4

**Table S1.** Photophysical Data for **Lipi-QA**, the Molecules **1**–**2**, and the Representative Long Fluorescence Lifetime Probes in Solutions

| Molecule | Solvent | $\lambda_{abs}$ / nm[a] | $\varepsilon$ / $M^{-1}$ $cm^{-1}$ | $\lambda_{em}$ / nm | $\Phi_F$[b] | **$\varepsilon \times \Phi_F$ / $M^{-1}$ $cm^{-1}$** | **$\tau$ / ns** |
|---|---|---|---|---|---|---|---|
| Lipi-QA | $CH_2Cl_2$ | 526 | 14600 | 542 | 0.98 | **14000** | **21.8** |
| **1** | $CH_2Cl_2$ | 520 | 18500 | 534 | 0.91 | **17000** | **19.8** |
| **2** | $CH_2Cl_2$ | 527 | 15600 | 543 | 0.99 | **15000** | **21.1** |
| ADOTA **1**[c] | DMSO | 540 | 11200 | 565 | 0.67 | **7500** | **19.3** |
| CDOTA[d] | $CH_2Cl_2$ | 535 | 9470 | 554 | 0.54 | **5100** | **23.1** |
| KU530[e] | $CH_3OH$ | 530 | 9800 | 560 | 0.60 | **5900** | **24** |
| SeTau-425[f] | PBS | 425 | 4200 | 545 | 0.39 | **1600** | **26.2** |

[a] The longest-wavelength absorption maximum.
[b] Absolute fluorescence quantum yield determined by a calibrated integrating sphere system.
[c] Reference: *Methods Appl. Fluoresc.*, **2015**, *3*, 045001.
[d] Reference: *Chem. Sci.*, **2018**, *9*, 3122.
[e] Reference: https://www.ku-dyes.com/ku-dyes-home/ku-dyes-catalogue/ku530-2/
[f] Reference: https://www.setabiomedicals.com/K7-545.html

**Table S2.** Photophysical Data for **Lipi-QA** in Various Solutions

| Solvent | $\lambda_{abs}$ / nm[a] | $\lambda_{em}$ / nm | $\Phi_F$[b] |
|---|---|---|---|
| $CH_2Cl_2$ | 526 | 542 | 0.98 |
| Toluene | 520 | 533 | 0.80 |
| $CHCl_3$ | 530 | 544 | 0.98 |
| $CH_3CN$ | 524 | 539 | 0.70 |
| PBS/DMSO (V/V 3/7) | 541 | 562 | 0.67 |
| Oleic Acid | 532 | 560 | 0.66 |

[a] The longest-wavelength absorption maximum.

[b] Absolute fluorescence quantum yield determined by a calibrated integrating sphere system.

**Table S3.** Comparison of the Super-Resolution Imaging of LDs Reported in the Literatures

| Year | Super-resolution imaging technique | Fluorescent probe | **FWHM resolution of image** | Fluorescence lifetime of probe | Time-gated detection (gate time) | Reference |
|---|---|---|---|---|---|---|
| 2019 | SIM | NIM-7 | – | – | No | 1 |
| 2021 | SIM | LD-FG | **180 nm** | – | No | 2 |
| 2019 | PALM | SNile Red | **<250 nm** | – | No | 3 |
| 2019 | STED | CM2P | **~300 nm** | – | No | 4 |
| 2021 | STED | LAQ1 | **166 nm** | – | No | 5 |
| 2021 | STED | DTPA-BT-M | **95 nm** | 4.73 ns | No | 6 |
| 2021 | STED | BoCz-Lip | **92 nm** | – | No | 7 |
| 2021 | STED | Lipi-DSB | **58 nm** | 1.98 ns | No | 8 |
| **2022** | **STED** | **Lipi-QA** | **37 nm** | **21.8 ns** | **Yes (6 ns)** | **This work** |

References:
[1] *Chem. Sci.* **2019**, *10*, 2342.
[2] *Angew. Chem. Int. Ed.* **2021**, *60*, 25104.
[3] *J. Am. Chem. Soc.* **2019**, *141*, 14699.
[4] *Anal. Chem.* **2019**, *91*, 977.
[5] *ACS Mater. Lett.* **2021**, *3*, 42.
[6] *Mater. Chem. Front.* **2021**, *5*, 1872.
[7] *Biosens. Bioelectron.* **2021**, *175*, 112871.
[8] *ACS Mater. Lett.* **2021**, *3*, 516.

**Table S4.** State-of-the-Art Performance of STED Super-Resolution Imaging Employing Various Organic Fluorescent Probes

| Year | Fluorescent probe | Cellular organelle | **Saturation intensity** | **FWHM resolution of image** | STED power (wavelength) | **Time-gated detection (gate time)** | Reference |
|---|---|---|---|---|---|---|---|
| 2019 | Mito-PB Yellow | mitochondria | **12.3 MW cm$^{-2}$** | **58 nm** | 270 mW (660 nm) | **Yes (3 ns)** | 1 |
| 2020 | MitoESq-635 | mitochondria | **4.37 MW cm$^{-2}$** | **35.2 nm** | 8.96 mW (775 nm) | **No** | 2 |
| 2021 | PID-CN | mitochondria | **4.7 MW cm$^{-2}$** | **70.9 nm** | 99.75 MW cm$^{-2}$ (775 nm) | **No** | 3 |
| 2022 | 2 | mitochondria | – | **93 nm** | – (775 nm) | **No** | 4 |
| 2022 | DTPAP-P | mitochondria | **8.7 MW cm$^{-2}$** | **165 nm** | – (660 nm) | **No** | 5 |
| 2014 | SiR-tubulin | tubulins | – | **39 nm** | – (775 nm) | **No** | 6 |
| 2017 | PB430-IgG | tubulins | – | **76 nm** | 30 mW (592 nm) | **Yes (0.5 ns)** | 7 |
| 2018 | SNIFP | vimentins | – | **73 nm** | – (860 nm) | **No** | 8 |
| 2020 | emiRFP703 | vimentins | – | **41 nm** | 25 mW (775 nm) | **No** | 9 |
| 2020 | Lyso-PB Yellow | lysosome | **1.0 MW cm$^{-2}$** | **70 nm** | 270 mW (660 nm) | **Yes (3 ns)** | 10 |
| 2021 | PIZ-CN | lysosome | **1.2 MW cm$^{-2}$** | **69 nm** | 2.75 MW cm$^{-2}$ (660 nm) | **No** | 3 |
| 2022 | DTPA-BT-F | lysosome | **11 MW cm$^{-2}$** | **107 nm** | 96 mW (775 nm) | **No** | 11 |
| 2015 | SiR-Hoechst | nuclear DNA | – | **78 nm** | – (775 nm) | **Yes (0.3 ns)** | 12 |

| | | | | | | | |
|---|---|---|---|---|---|---|---|
| 2021 | α-Lamin-Atto594 | nuclear lamina | – | **40 nm** | – | **No** | 13 |
| 2020 | Dil-SiR | ER tubules | – | **44 nm** | – (775 nm) | – | 14 |
| **2022** | **Lipi-QA** | **lipid droplets** | **1.6 MW cm$^{-2}$** | **37 nm** | **5 MW cm$^{-2}$ (660 nm)** | **Yes (6 ns)** | **This work** |

Reference:
[1] *Proc. Natl. Acad. Sci. U.S.A.* **2019**, *116*, 15817.
[2] *Nat. Commun.* **2020**, *11*, 3699.
[3] *Adv. Funct. Mater.* **2021**, *31*, 2009329.
[4] *Angew. Chem. Int. Ed.* **2022**, *61*, e202111855.
[5] *ACS Nano* **2022**, doi.org/10.1021/acsnano.1c11125.
[6] *Nat. Methods* **2014**, *11*, 731.
[7] *J. Am. Chem. Soc.* **2017**, *139*, 10374.
[8] *Nat. Commun.* **2018**, *9*, 4762.
[9] *Nat. Commun.* **2020**, 11, 239.
[10] *ACS Mater. Lett.* **2020**, *2*, 705.
[11] *Chem. Sci.* **2022**, *13*, 1270.
[12] *Nat. Commun.* **2015**, *6*, 8497.
[13] *Angew. Chem. Int. Ed.* **2021**, *60*, 22075.
[14] *Nat. Commun.* **2020**, *11*, 4271.

## 4. Supplementary Figure S1 to S17

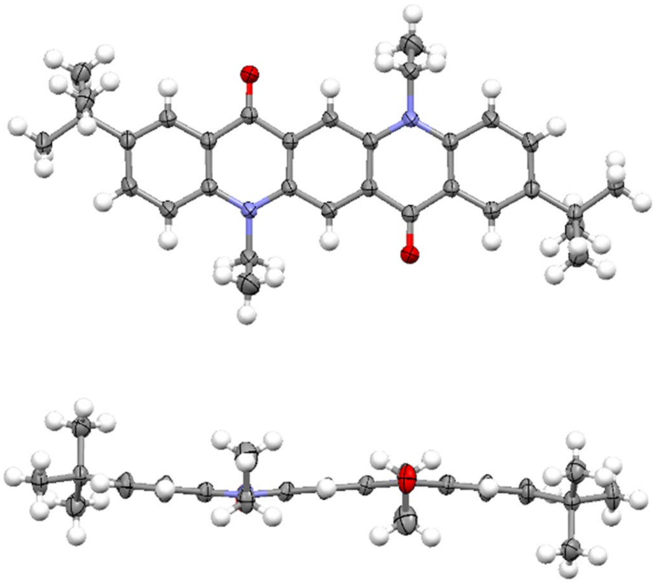

**Figure S1.** Top view and side view of the X-ray single crystal structure of **Lipi-QA**.

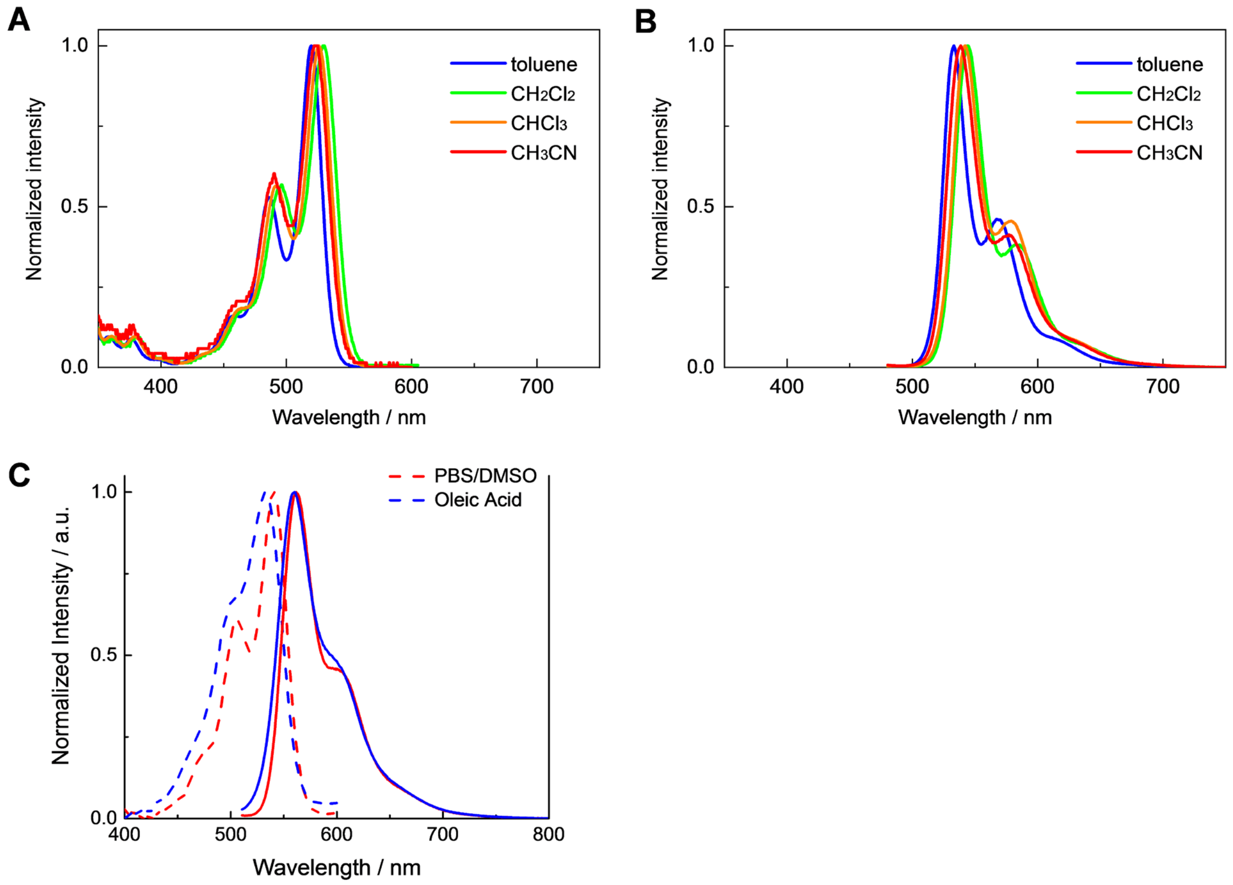


**Figure S2.** Normalized (A) absorption and (B) emission spectra of the fluorescent probe **Lipi-QA** in various organic solvents; (C) Normalized absorption and emission spectra of **Lipi-QA** in the aqueous mixture of PBS/DMSO (V/V = 3/7) and oleic acid.

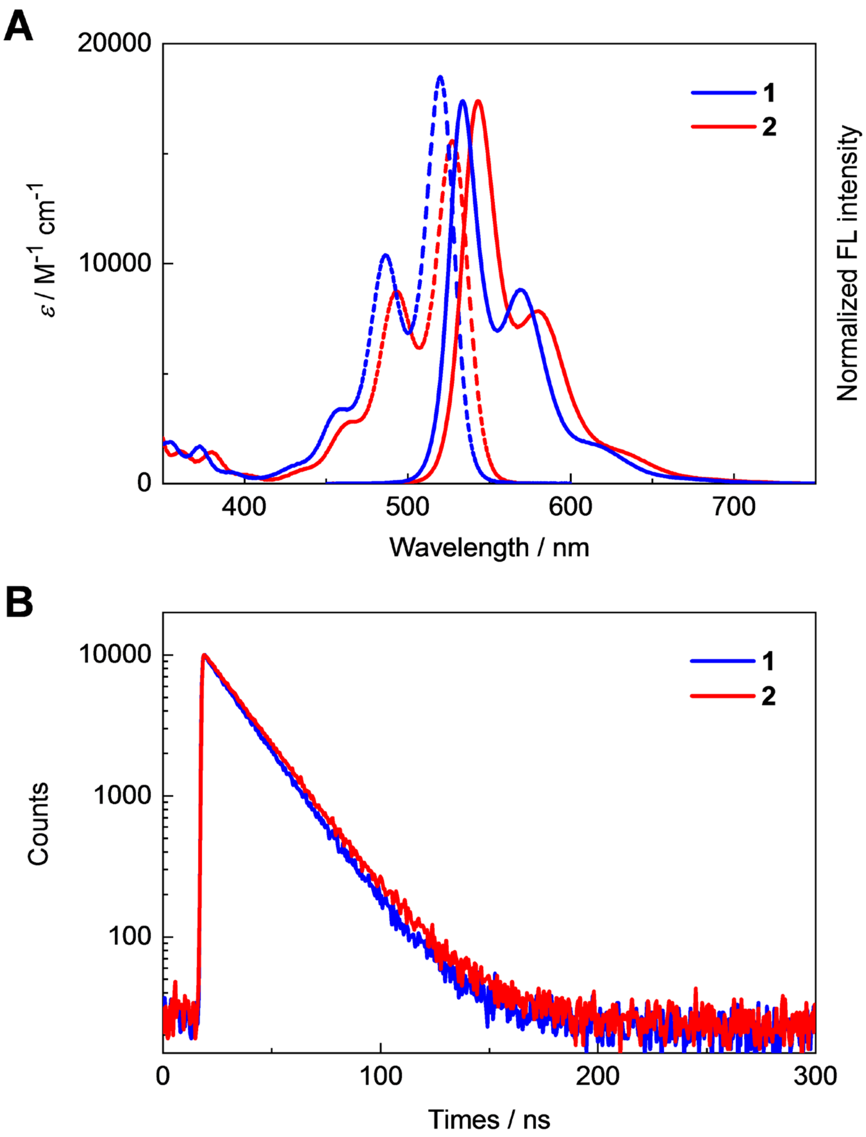


**Figure S3.** Photophysical properties of the molecules **1**–**2** in $CH_2Cl_2$ solutions: (A) absorption (dashed lines) and fluorescence (solid lines) spectra; (B) fluorescence decay profiles.

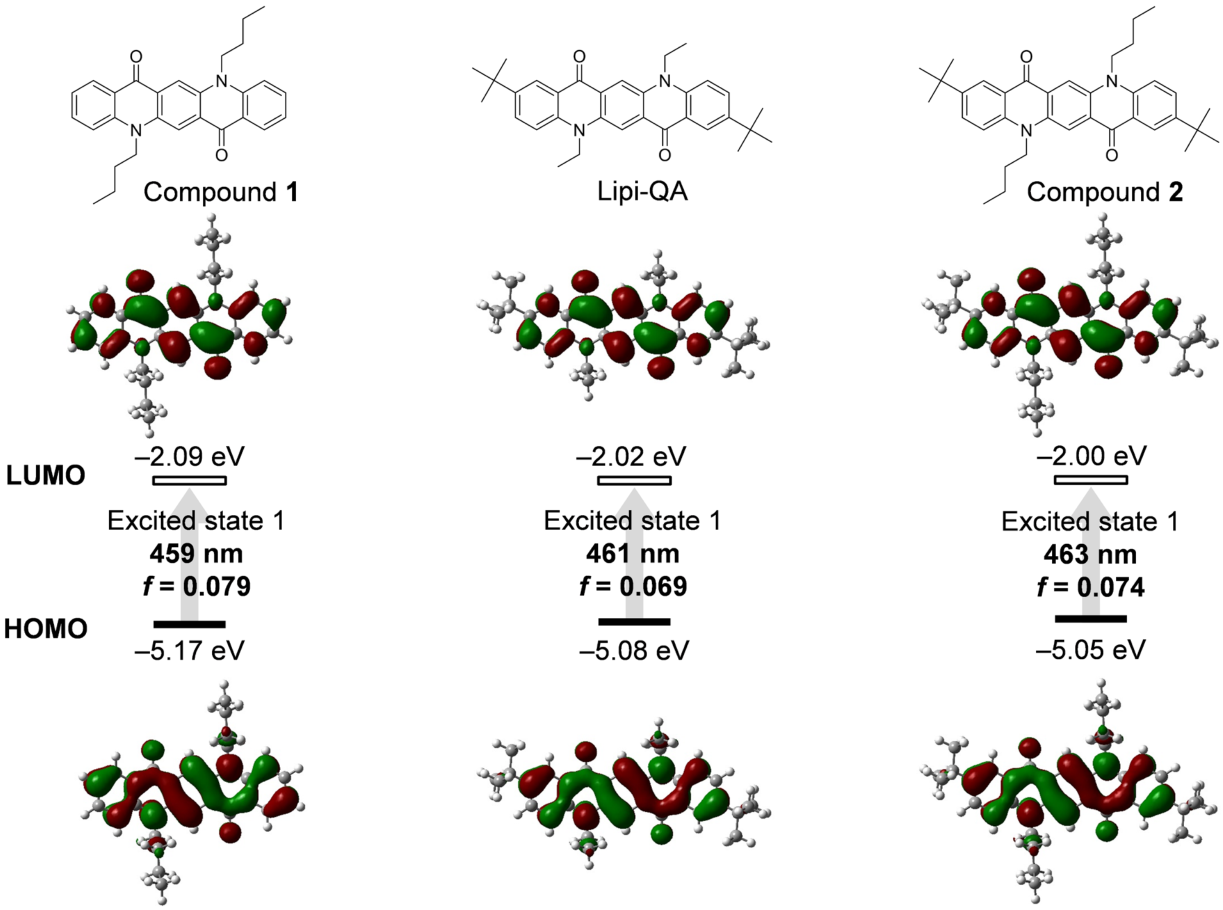


**Figure S4.** TD-DFT calculation (B3LYP/6-31G*, Gaussian 09) results of **Lipi-QA**, compound **1** and **2**: energy diagram, Kohn−Sham HOMO and LUMO, vertical excitation wavelength, and oscillator strengths (*f*). The first excited state corresponds to the transition of HOMO to LUMO.

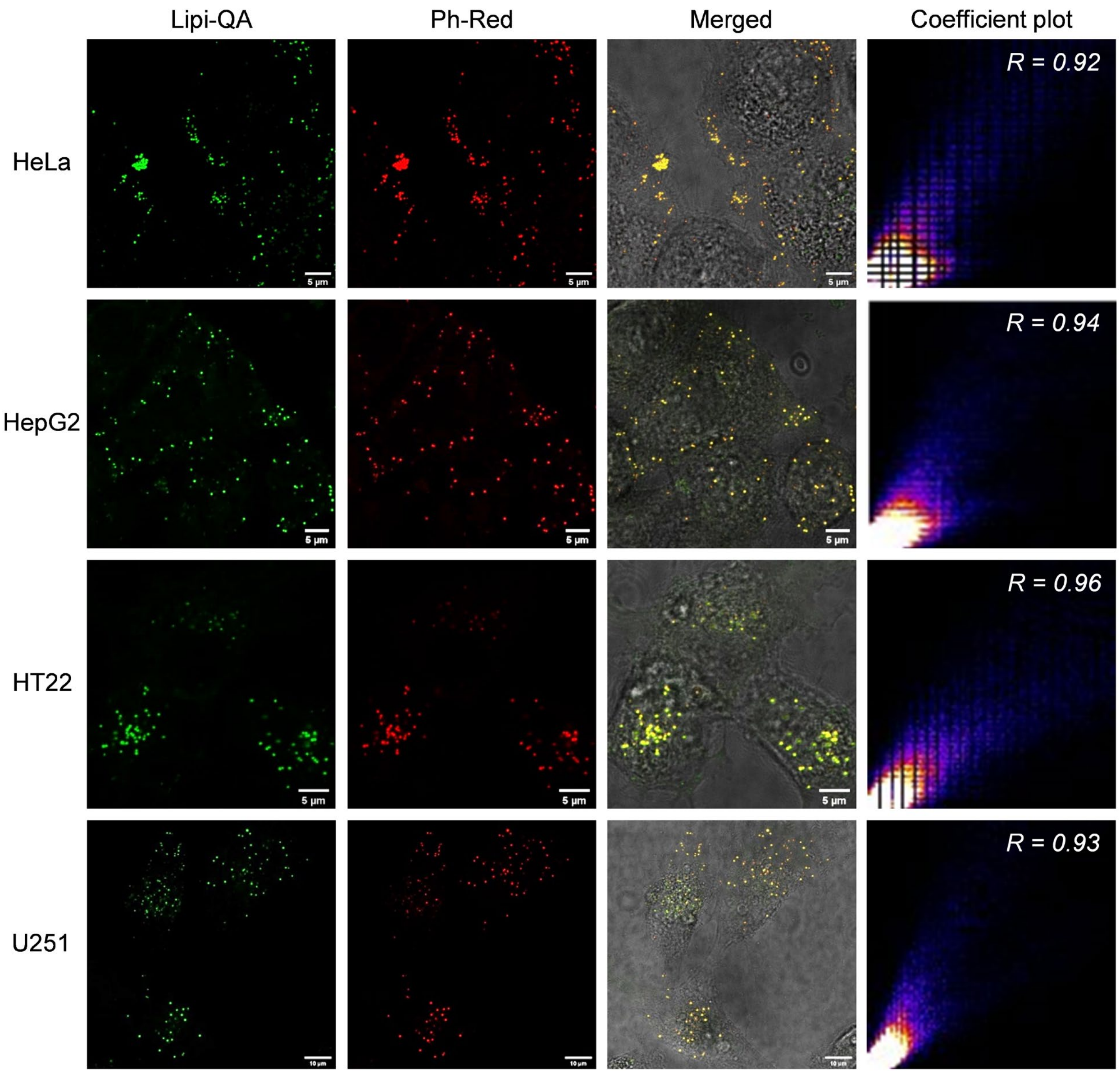


**Figure S5.** Co-localization confocal imaging of living cells (HeLa, HepG2, HT22, and U251) stained with **Lipi-QA** (2 μM, 2 h) and the lipid droplets fluorescent probe Ph-Red (1 μM, 2 h): the green imaging channel of Lipi-QA ($\lambda_{ex}$ = 488 nm, $\lambda_{em}$ = 500–540 nm); the red imaging channel of Rh-Red ($\lambda_{ex}$ = 488 nm, $\lambda_{em}$ = 680–720 nm); the merged image of two fluorescence channels and bright filed; the Pearson's correlation coefficient plot of the two fluorescence channels. Scale bar: 5 μm for HeLa, HepG2, and HT22; 10 μm for U251.

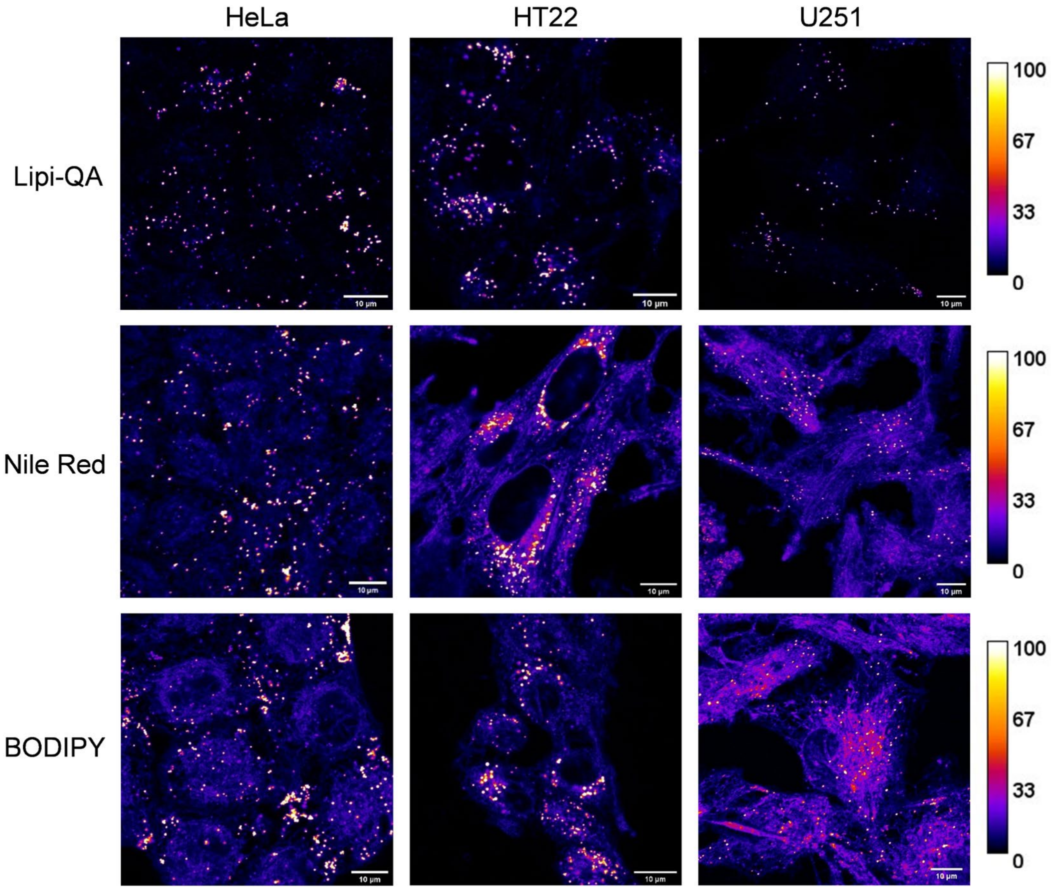


**Figure S6.** Comparison of the LDs staining specificity between the fluorescent probes **Lipi-QA**, Nile Red and BODIPY 493/503 (2 μM, 2 h) in living cells (HeLa, HT22, and U251). $\lambda_{ex}$ = 488 nm, $\lambda_{em}$ = 500–700 nm. Scale bar: 10 μm.

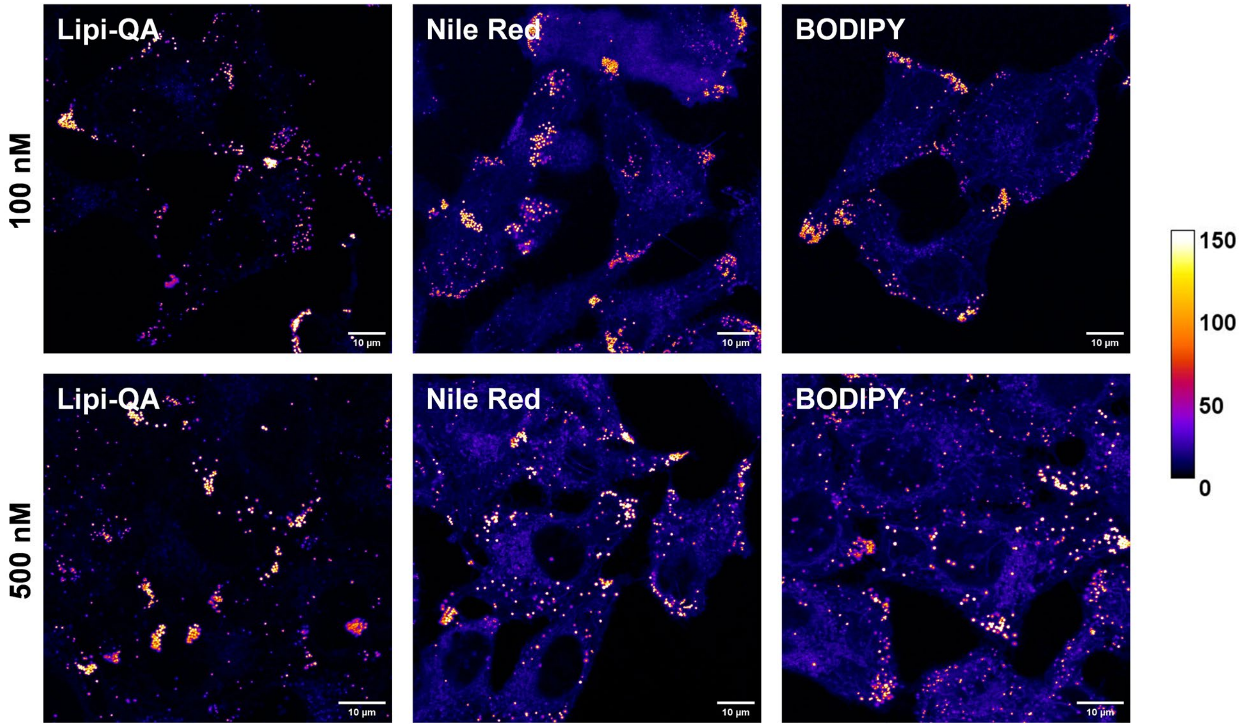


**Figure S7.** Comparison of the LDs staining specificity between the fluorescent probes **Lipi-QA**, Nile Red and BODIPY at the concentration of 100 nM and 500 nM (2 h) in living HeLa cells. $\lambda_{ex}$ = 488 nm, $\lambda_{em}$ = 500–700 nm. Scale bar: 10 μm.

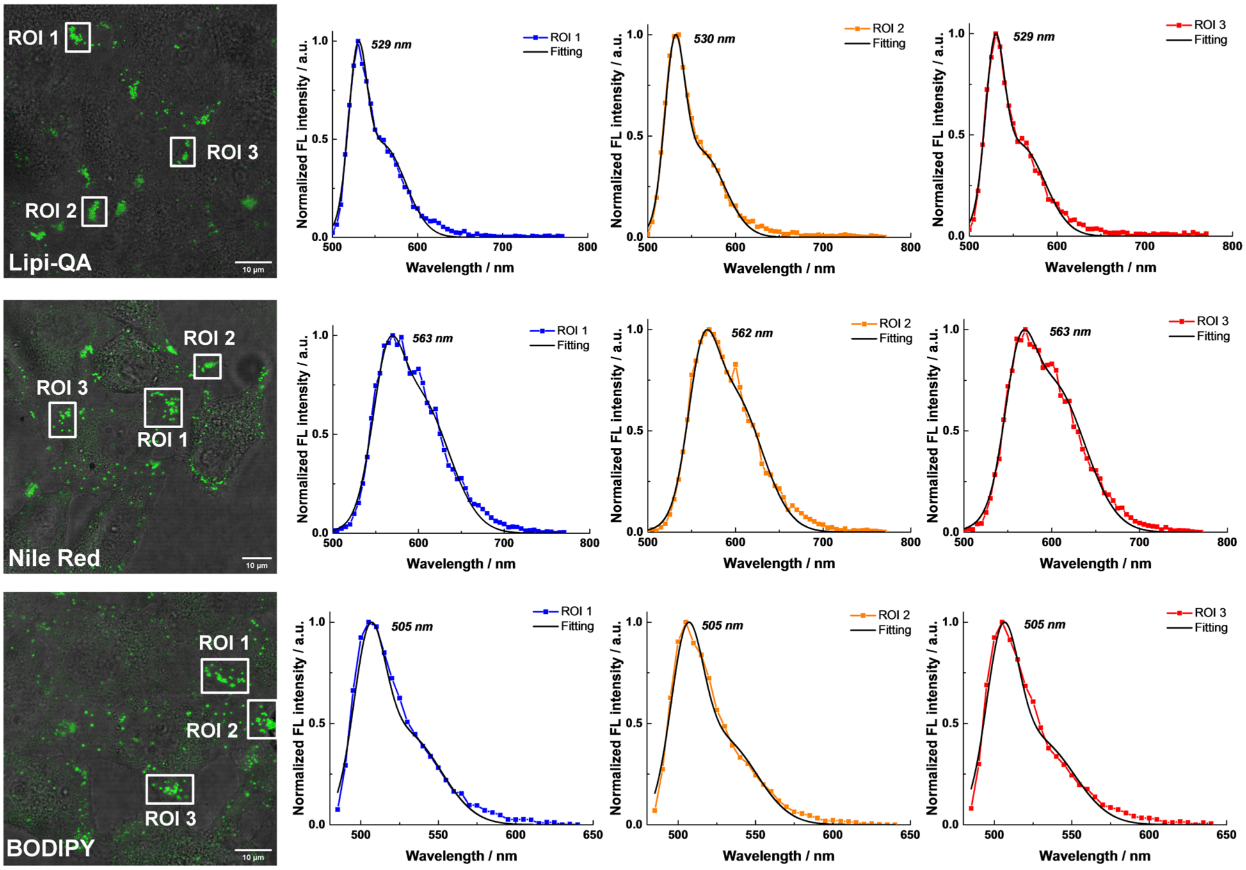


**Figure S8.** Lambda scan imaging of **Lipi-QA**, Nile Red and BODIPY in HeLa cells (500 nM, 2 h). The excitation laser: 488 nm for **Lipi-QA** and Nile Red, 476 nm for BODIPY; detection range: 500-780 nm for **Lipi-QA** and Nile Red, 485-650 nm for BODIPY. The in-situ emission spectra of regions of interests (ROIs) are fitted with Gaussian function to provide the emission maxima. Scale bar: 10 μm.

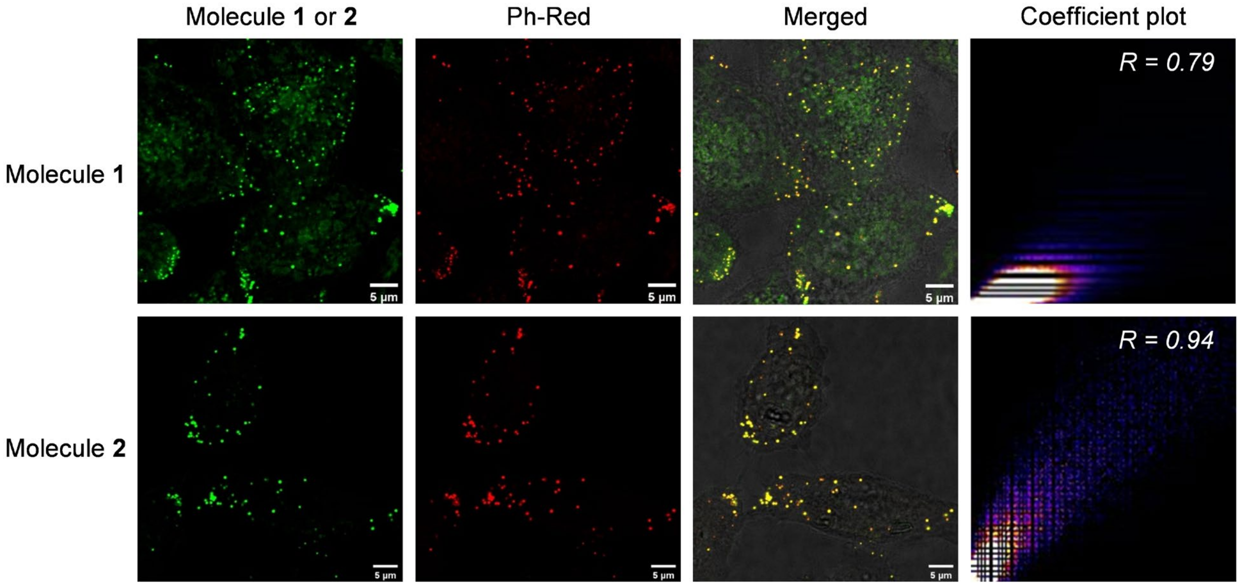


**Figure S9.** Co-localization confocal imaging of living HeLa cells stained with molecules **1** or **2** (2 μM, 2 h) and the LDs probe Ph-Red (1 μM, 2 h): the green imaging channel of molecules **1**–**2** ($\lambda_{ex}$ = 488 nm, $\lambda_{em}$ = 500–540 nm); the red imaging channel of Rh-Red ($\lambda_{ex}$ = 488 nm, $\lambda_{em}$ = 680–720 nm); the merged image of two fluorescence channels and bright filed; the Pearson's correlation coefficient plot of the two fluorescence channels ($R$ = 0.79 for molecules **1**; $R$ = 0.94 for molecules **2**). Scale bar: 5 μm.

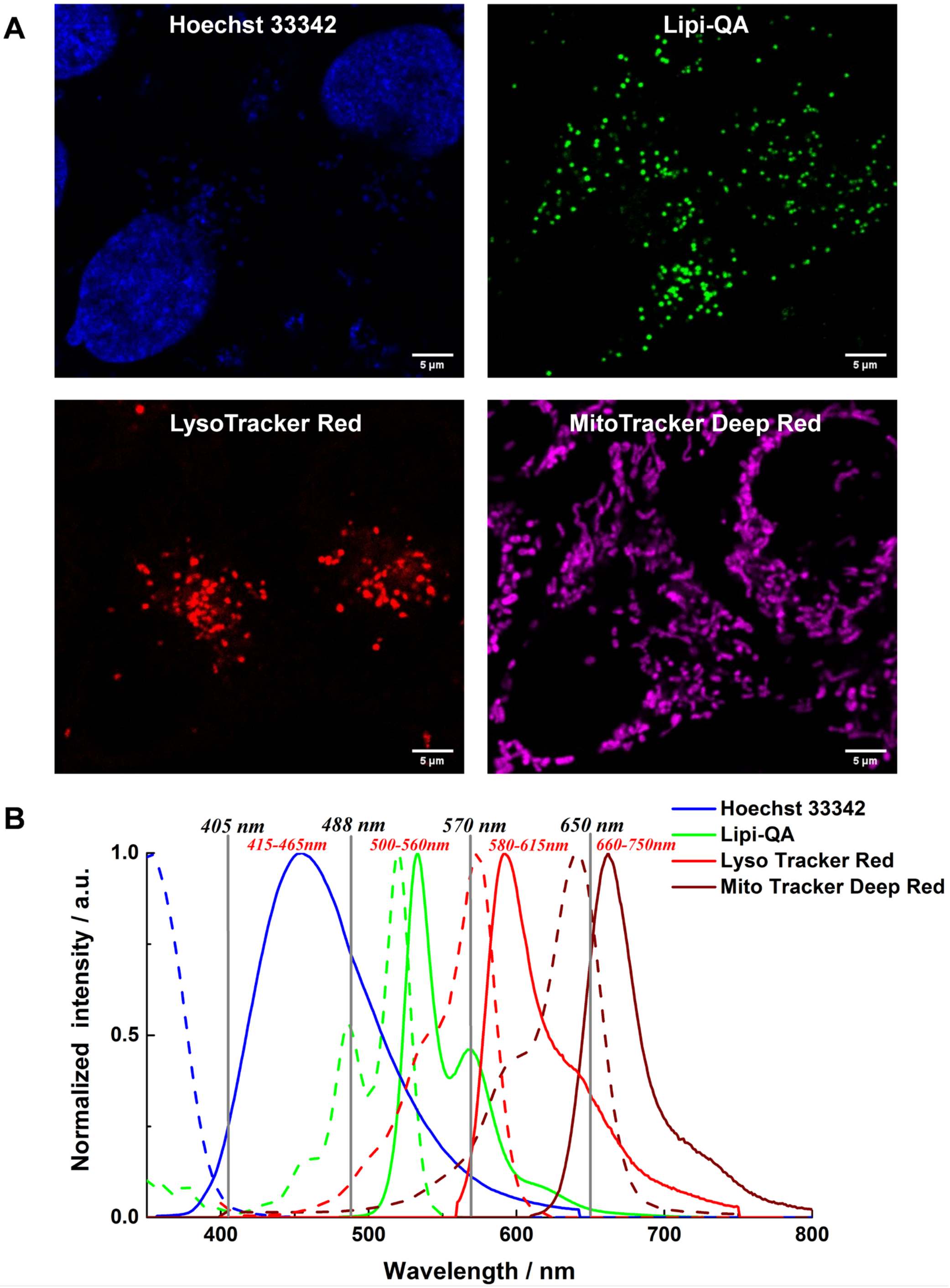


**Figure S10.** Multicolor confocal imaging of living HeLa cells: (A) the four imaging channels of Hoechst 33342 (20 µM, 0.5 h), **Lipi-QA** (2 µM, 2 h), LysoTracker Red (200 nM, 0.5 h), and MitoTracker Deep Red (50 nM, 0.5 h), respectively. Scale bar: 5 µm. (B) Absorption (dashed line) and fluorescence (solid line) spectra of the four probes used in the multicolor confocal imaging. The excitation wavelength and detection of emission range of each probe are indicated sequentially.

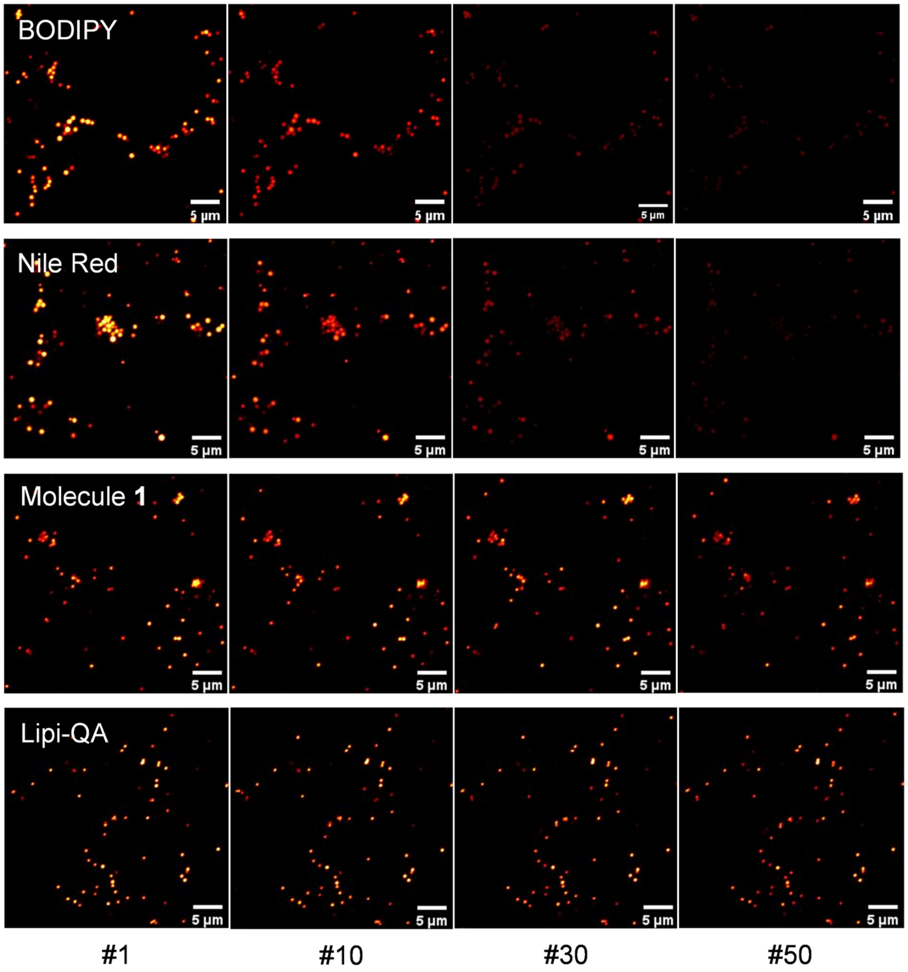


**Figure S11.** Comparison of the photostability between BODIPY 493/503, Nile Red, molecules **1**, and **Lipi-QA**. The confocal images of HeLa cells stained with these fluorescent probes (2 μM, 2 h) were repeatedly recorded under the identical and intense excitation condition ($\lambda_{ex}$ = 488 nm, about 100 times stronger laser power of the common imaging condition); the confocal images of number 1, 10, 30 and 50 were shown. $\lambda_{ex}$ = 488 nm, $\lambda_{em}$ = 500–700 nm. Scale bar: 5 μm.

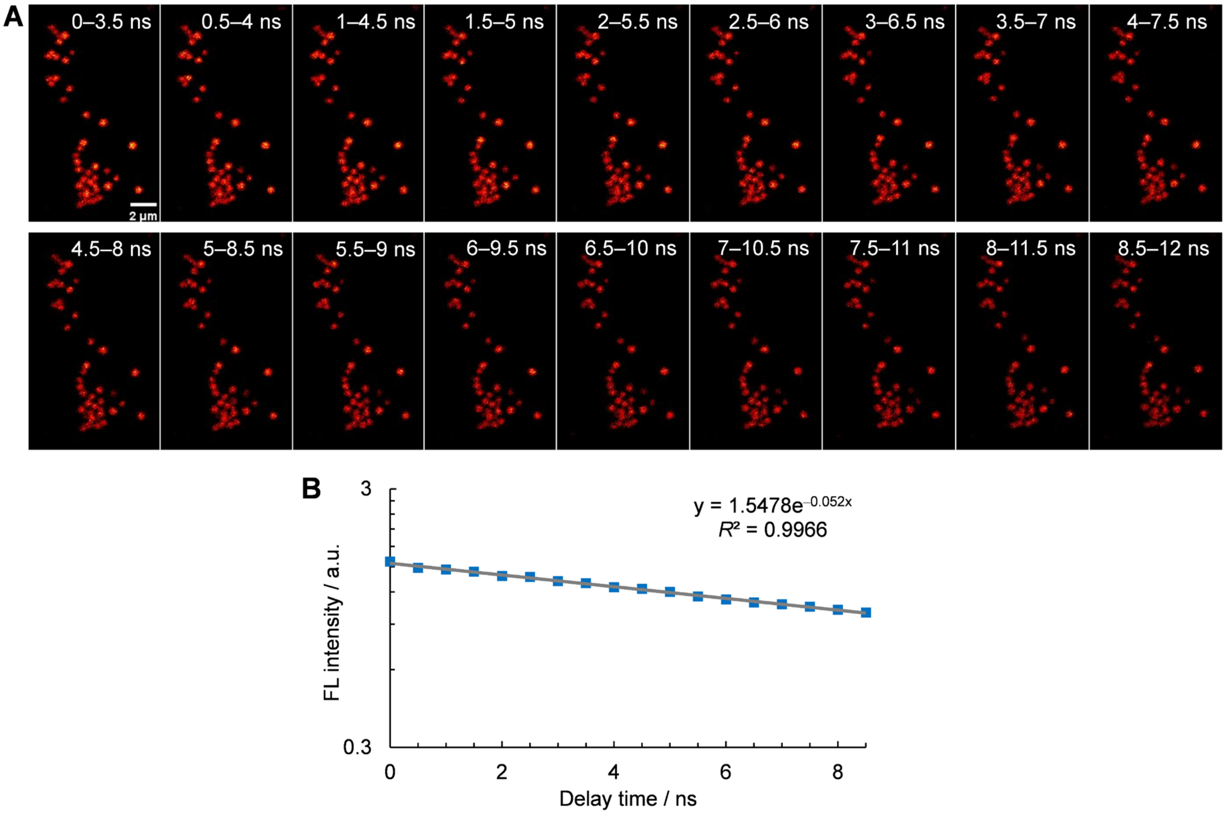


**Figure S12.** Determination of the fluorescence lifetime of **Lipi-QA** in LDs: (A) the time-gated confocal images of LDs stained with **Lipi-QA** (2 μM, 2 h); the images were collected during the gated time indicated ($\Delta t$ = 3.5 ns); $\lambda_{ex}$ = 488 nm, $\lambda_{em}$ = 500–700 nm. Scale bar: 2 μm; (B) the fluorescence intensities of images were plotted as a function of the delay time (blue spots); the fluorescence lifetime of **Lipi-QA** was determined by a single-exponential-decay fitting (gray line).

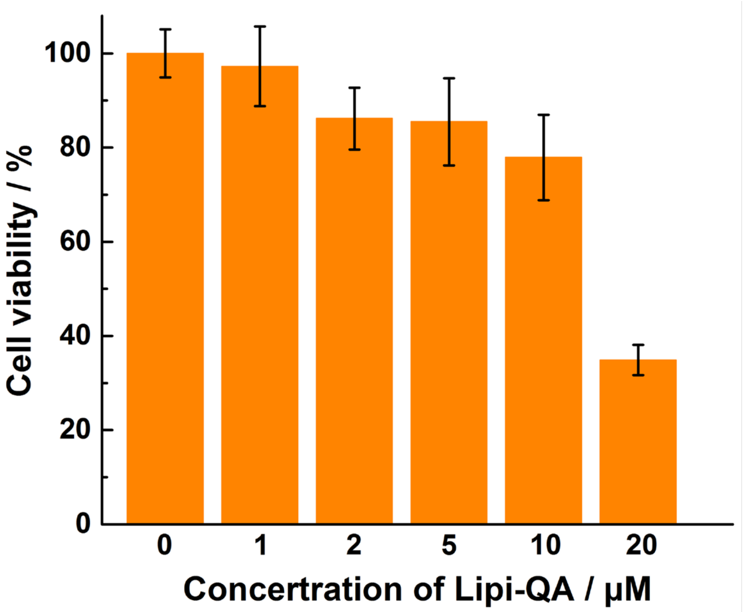


**Figure S13.** Cell viability results of the HeLa cells stained with **Lipi-QA** by MTT assay. The results are expressed as percentages of the dye-free controls. All data are presented as mean ± s.d. ($n$ = 10).

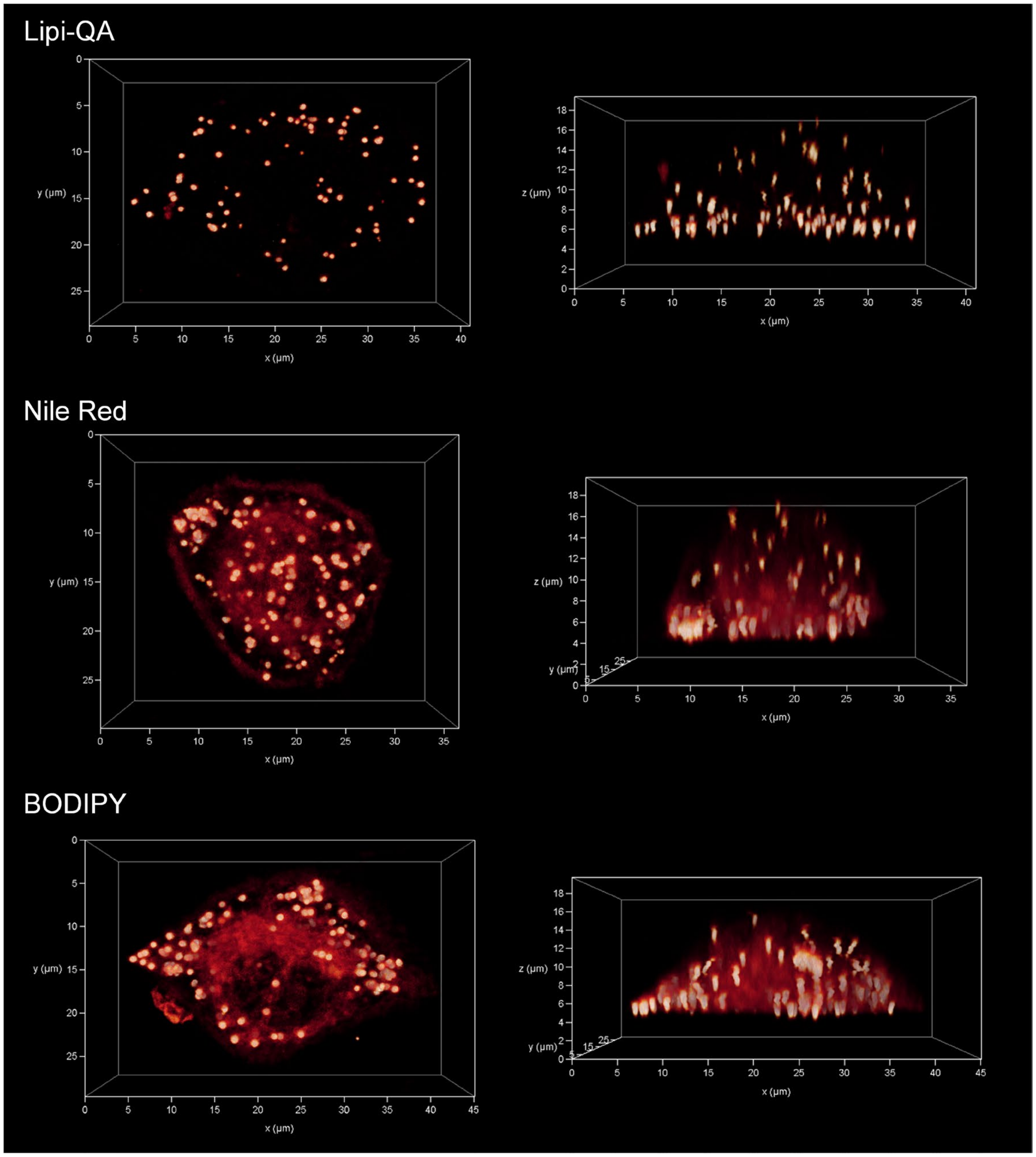


**Figure S14.** 3D confocal imaging (top view and side view) of living HeLa cells stained with **Lipi-QA**, Nile Red, and BODIPY (2 µM, 2 h), respectively. $\lambda_{ex}$ = 488 nm, $\lambda_{em}$ = 500–700 nm. The high LDs selectivity is particularly important to clearly visualize the spatial distribution of LDs.

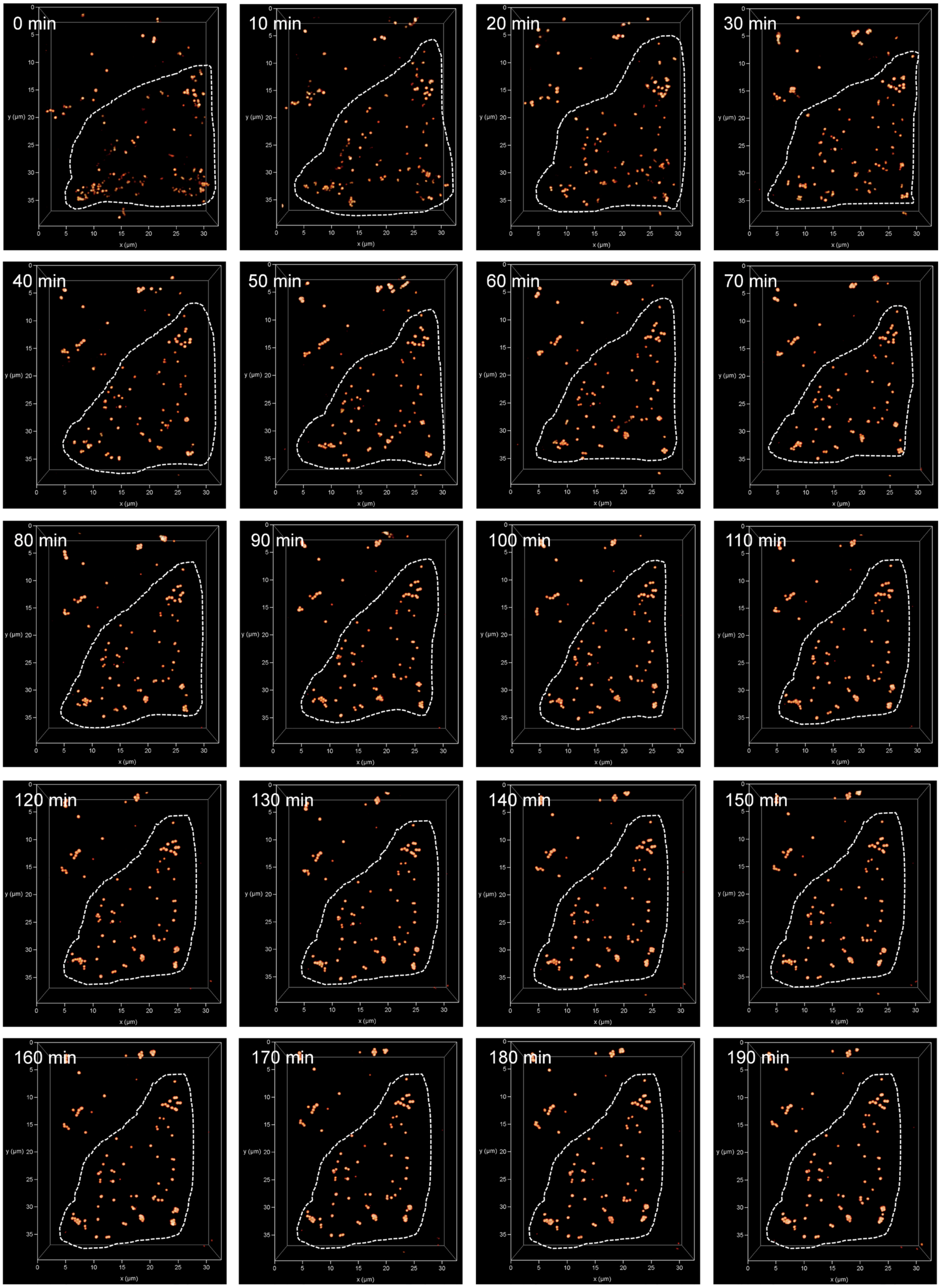


**Figure S15.** Time-lapse three-dimensional (3D) confocal imaging of living HeLa cells labeled with **Lipi-QA** (2 μM, 2 h) under starvation stimulation. $\lambda_{ex}$ = 488 nm, $\lambda_{em}$ = 500–700 nm.

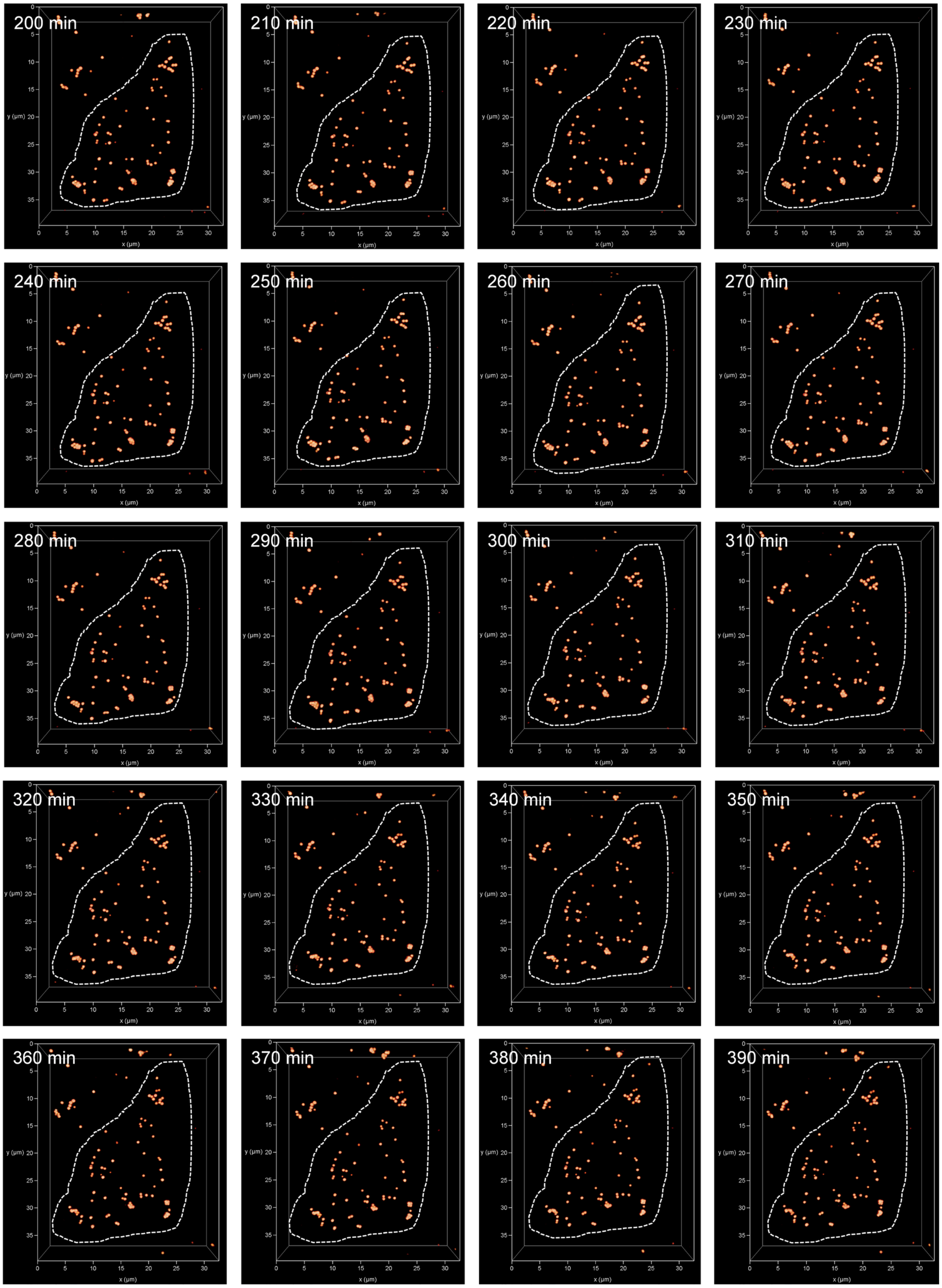


**Figure S15. (continue)** Time-lapse three-dimensional (3D) confocal imaging of living HeLa cells labeled with **Lipi-QA** (2 μM, 2 h) under starvation stimulation. $\lambda_{ex}$ = 488 nm, $\lambda_{em}$ = 500–700 nm.

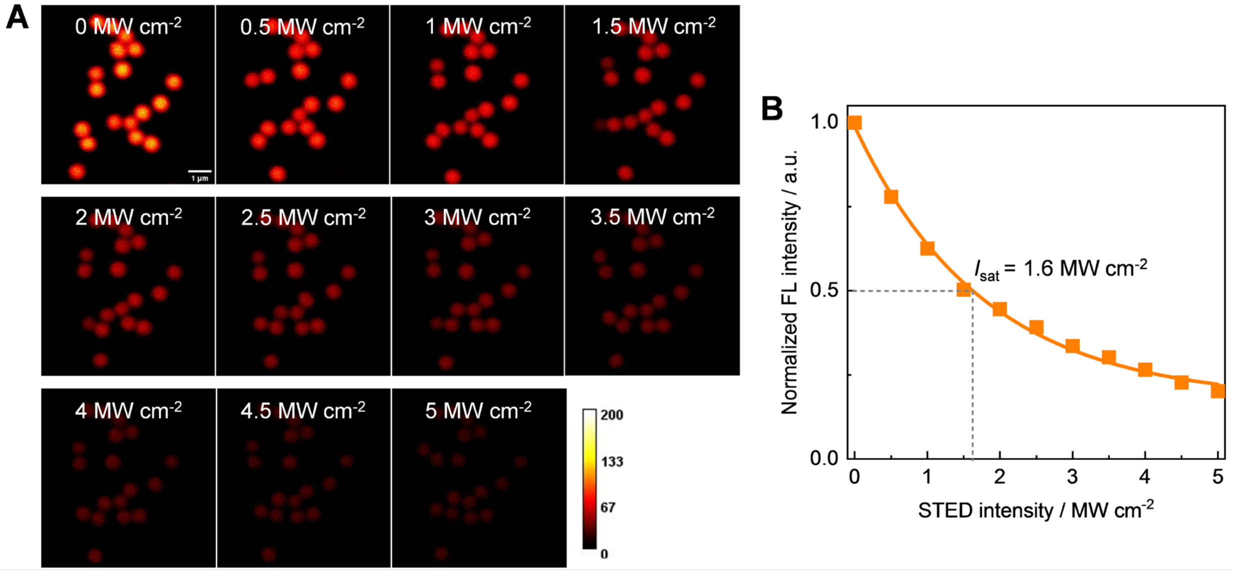


**Figure S16.** Determination of the saturation intensity ($I_{sat}$) of **Lipi-QA** under 660 nm CW-STED laser. (A) The fluorescence images of HeLa cells stained with **Lipi-QA** (2 μM, 2 h) under the identical excitation condition ($\lambda_{ex}$ = 488 nm) while gradually increasing the STED laser power from 0 to 5 MW cm$^{-2}$. (B) The fluorescence intensities of each images plotted as a function of the STED laser intensities.

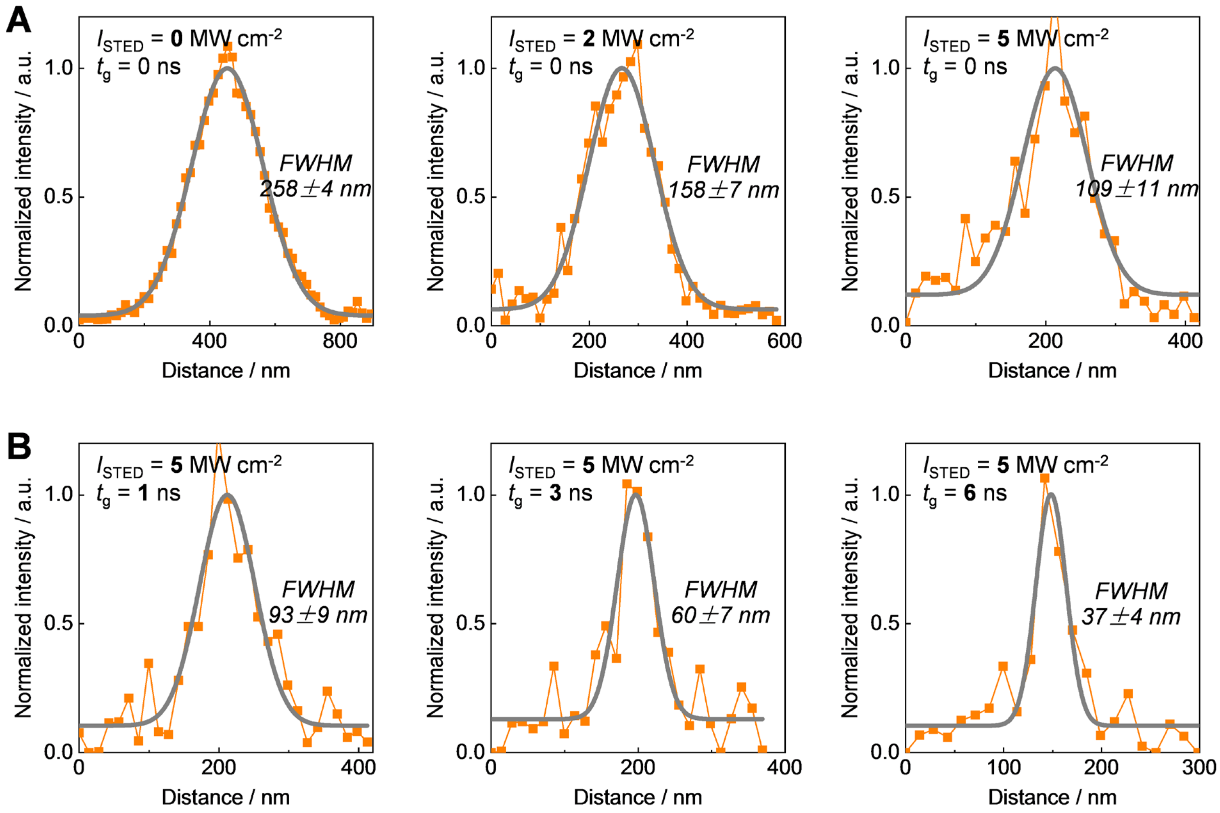


**Figure S17.** The STED laser intensity-dependent resolution and the gating detection delay time-dependent resolution. The signal intensity profiles crossed the LDs (shown in the insets of Figure 4A and Figure 4C) were fitted with Gaussian fitting (gray lines).

## 5. Discussion of the resolution

**Relationship between the $I_{sat}$ value and the resolution.** According to the STED super-resolution imaging theory, the resolution $\Delta r$ is determined by the following equation in principle:

$$\Delta r \approx \frac{\lambda_{ex}}{2NA\sqrt{1+\frac{I_{STED}}{I_{sat}}}}$$

where $\lambda_{ex}$ is the excitation wavelength, $NA$ is the numerical aperture, $I_{STED}$ is the intensity of STED laser, and the $I_{sat}$ is the saturation intensity. In general, the $\lambda_{ex}$ and $NA$ are constants for a STED super-resolution microscopy. Therefore, decreasing the $I_{sat}$ value is highly desired to achieve high resolution.

**Relationship between the $I_{sat}$ value and the fluorescence lifetime.** According to the STED super-resolution imaging theory, the saturation intensity $I_{sat}$ is determined by the following equation:

$$I_{sat} = \frac{hc}{\sigma\tau\lambda_{STED}}$$

where $h$ is the Planck constant, $c$ is the speed of light, $\sigma$ is the cross section at the STED laser wavelength, $\tau$ is the fluorescence lifetime of molecule, $\lambda_{STED}$ is the wavelength of STED laser. Since the $h$, c, and $\lambda_{STED}$ are constants, increasing the $\tau$ value could directly decrease the $I_{sat}$ value and thus contribute to high resolution.

## 6. Captions for Movies S1 to S2

**Movie S1.** Time-lapse STED super-resolution imaging up to 500 STED frames

**Movie S2.** Time-lapse STED super-resolution imaging of a LD fission

## 7. NMR spectra of synthesized compounds

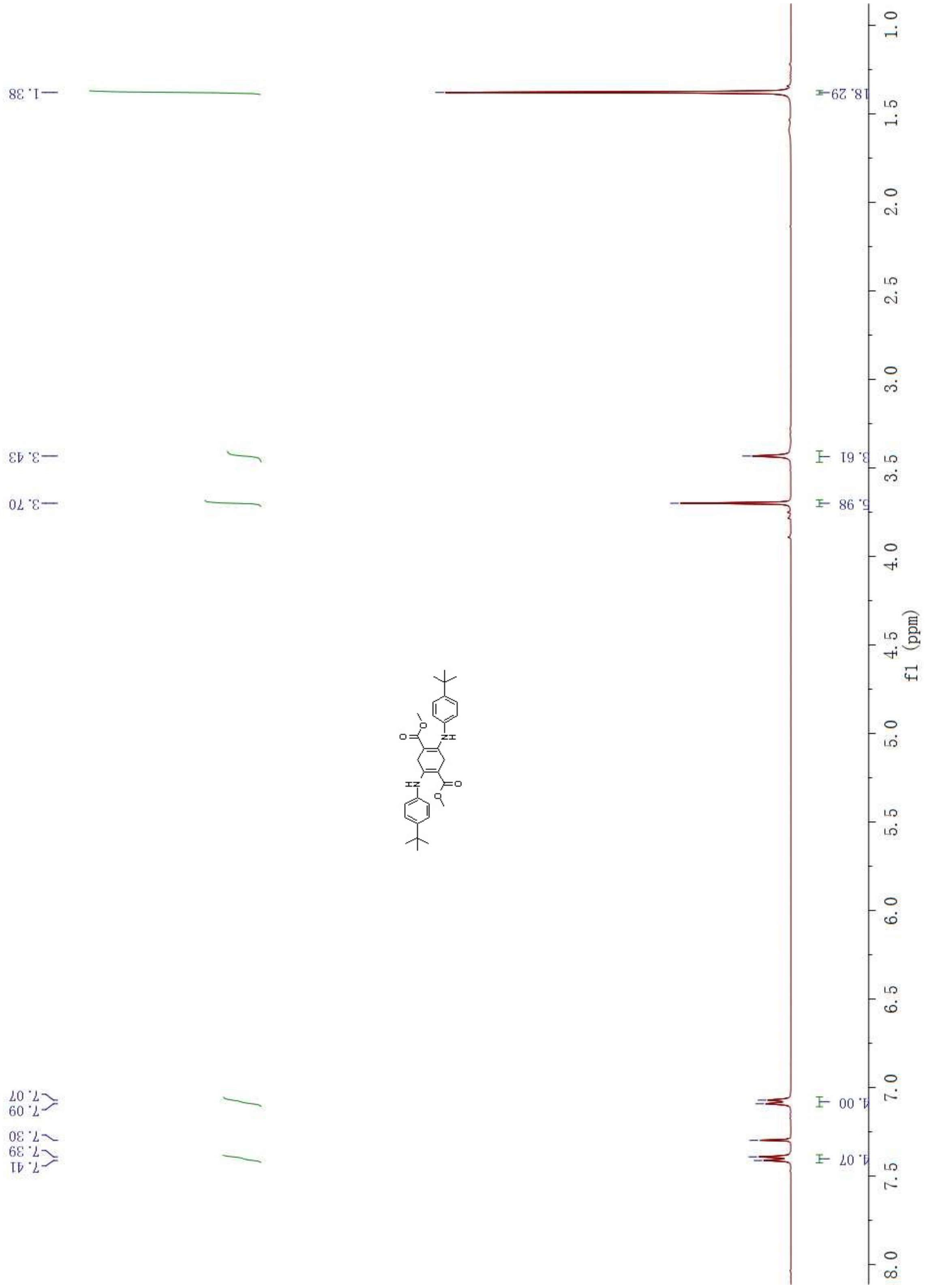

**NMR-1.** $^{1}H$ NMR spectrum of compound **4** (400 MHz, $CDCl_3$).

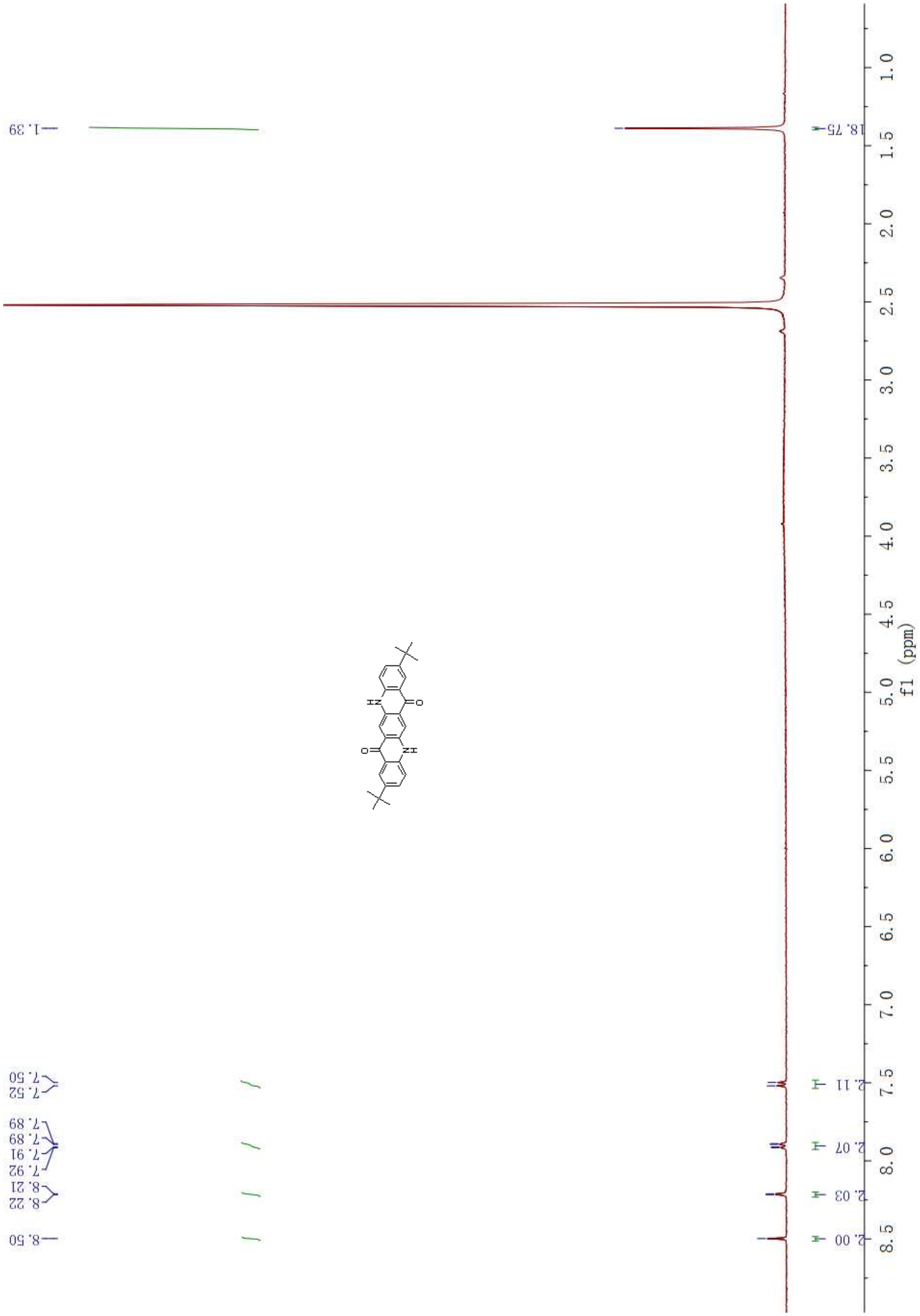


**NMR-2.** $^{1}$H NMR spectrum of compound **6** (400 MHz, DMSO-$d_6$).

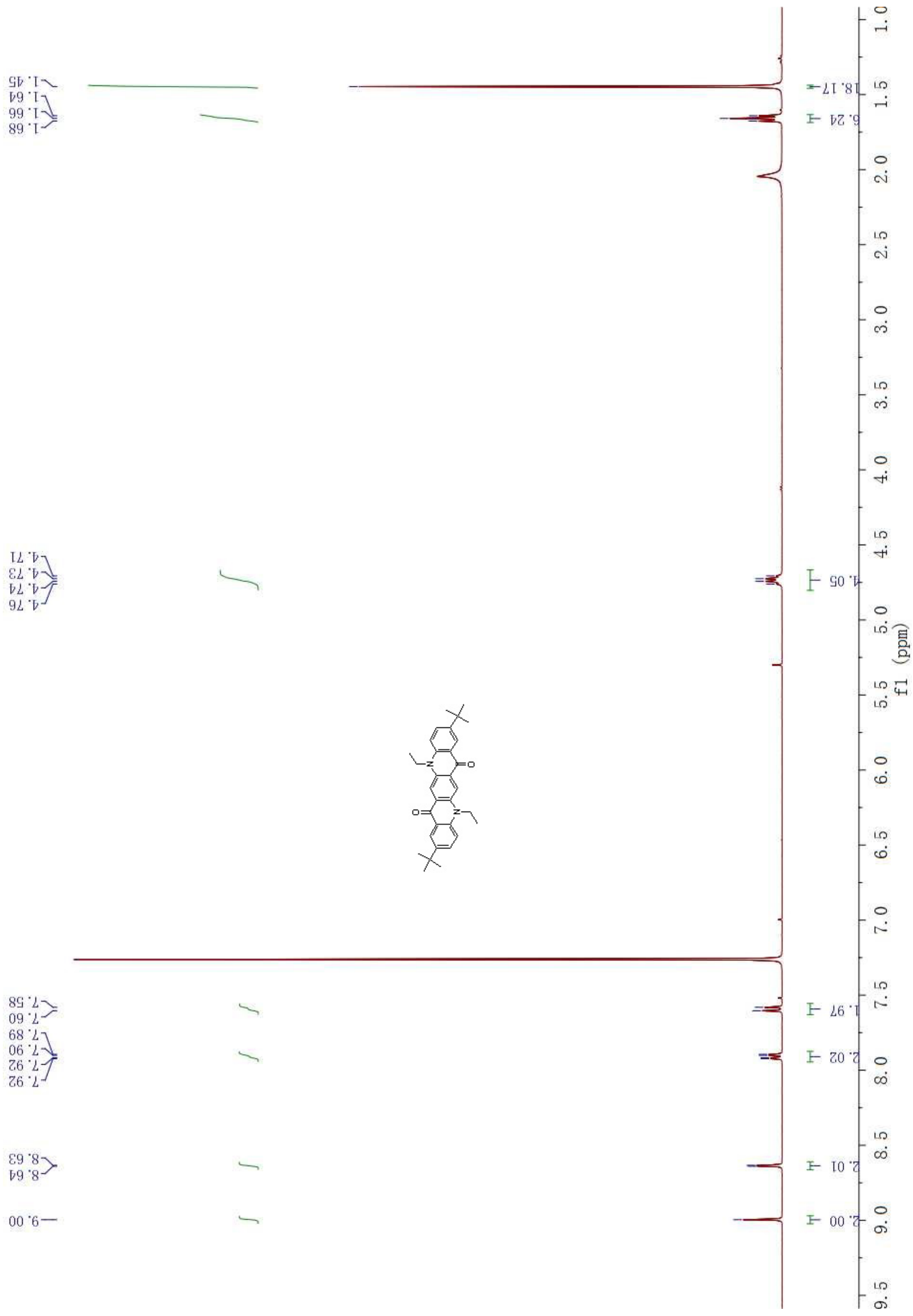


**NMR-3.** $^{1}$H NMR spectrum of **Lipi-QA** (400 MHz, $CDCl_3$).

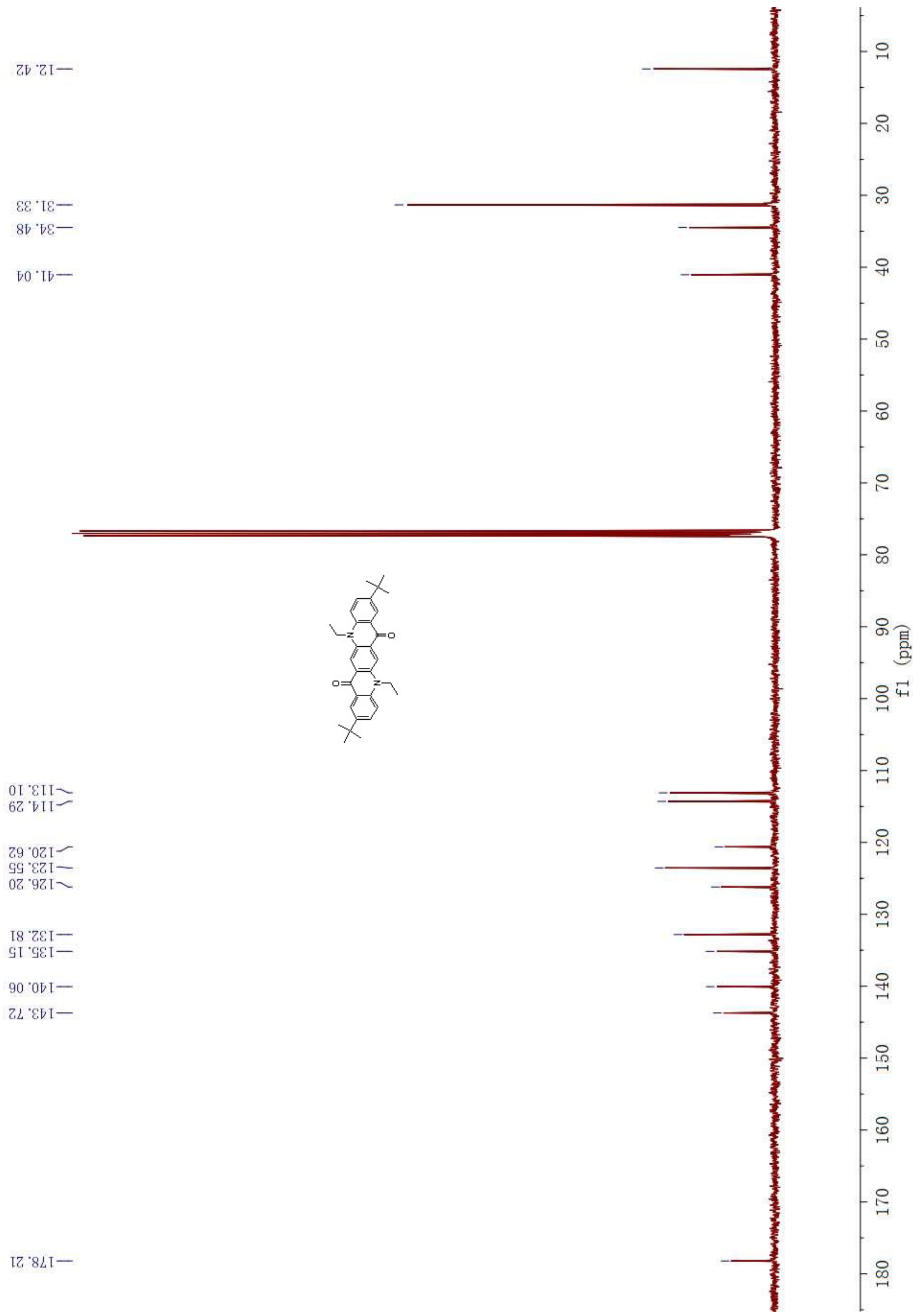


**NMR-4.** $^{13}$C NMR spectrum of **Lipi-QA** (101 MHz, $CDCl_3$).

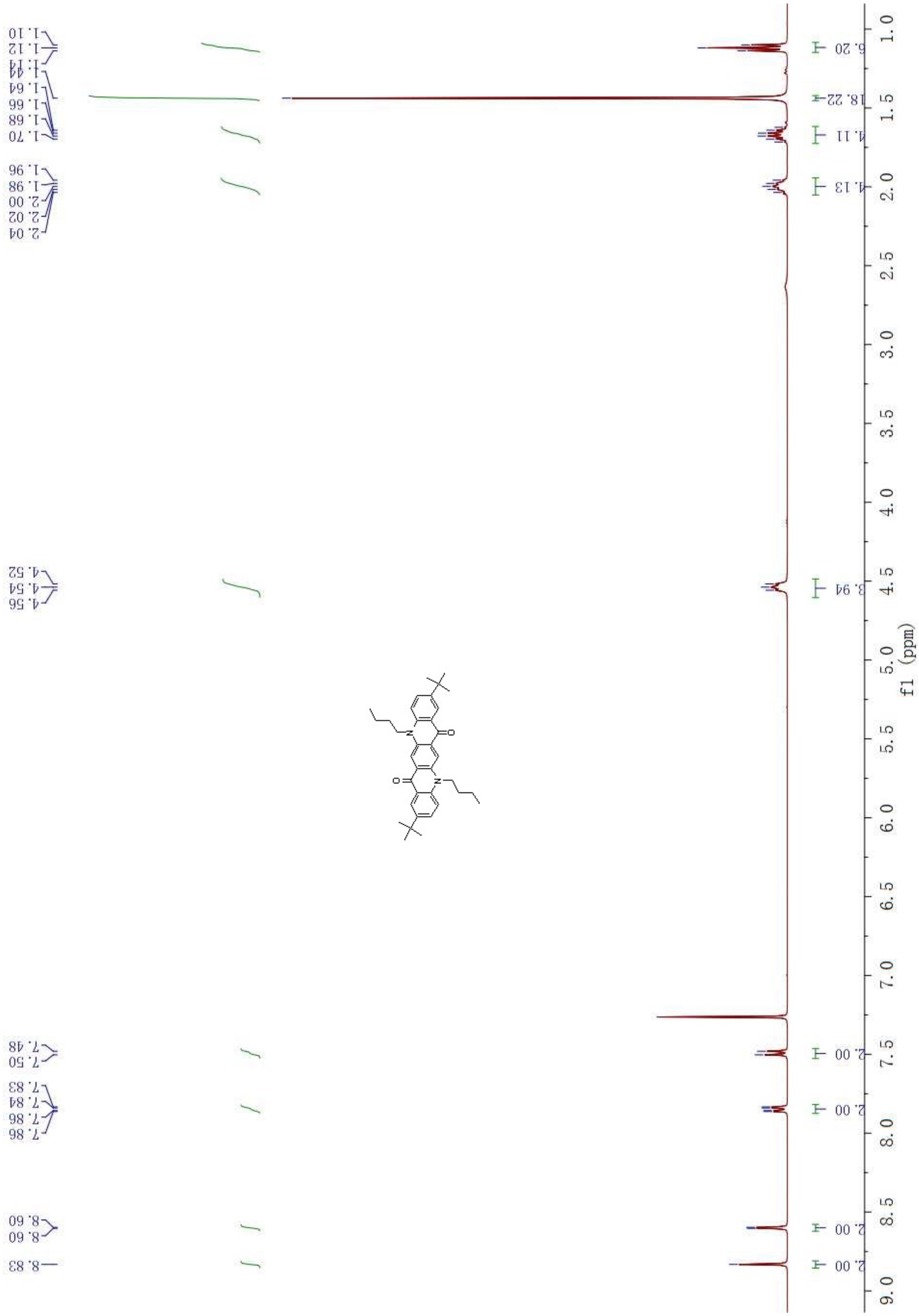


**NMR-5.** $^{1}$H NMR spectrum of compound **2** (400 MHz, $CDCl_3$).

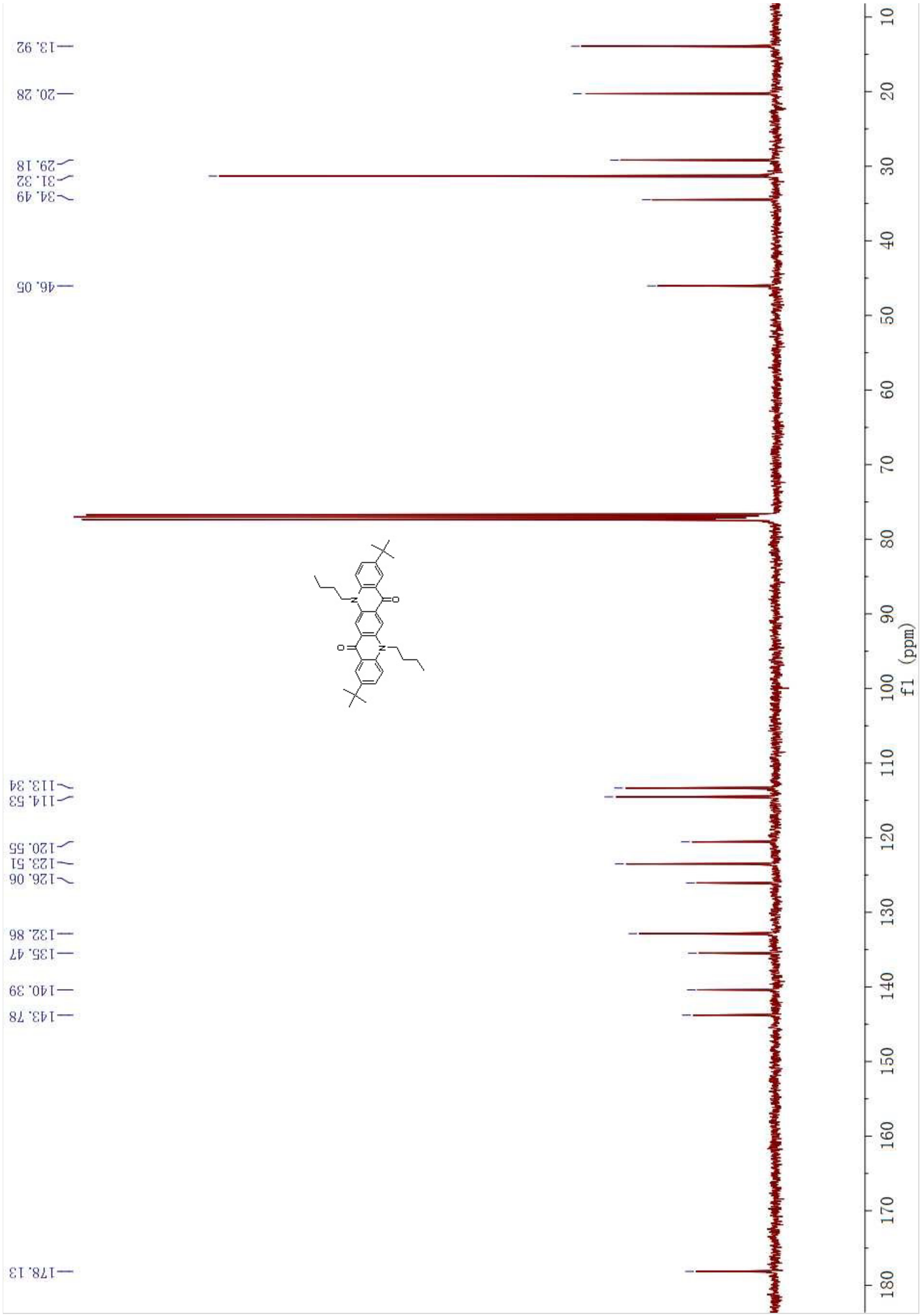

**NMR-6.** $^{13}$C NMR spectrum of compound **2** (101 MHz, $CDCl_3$).

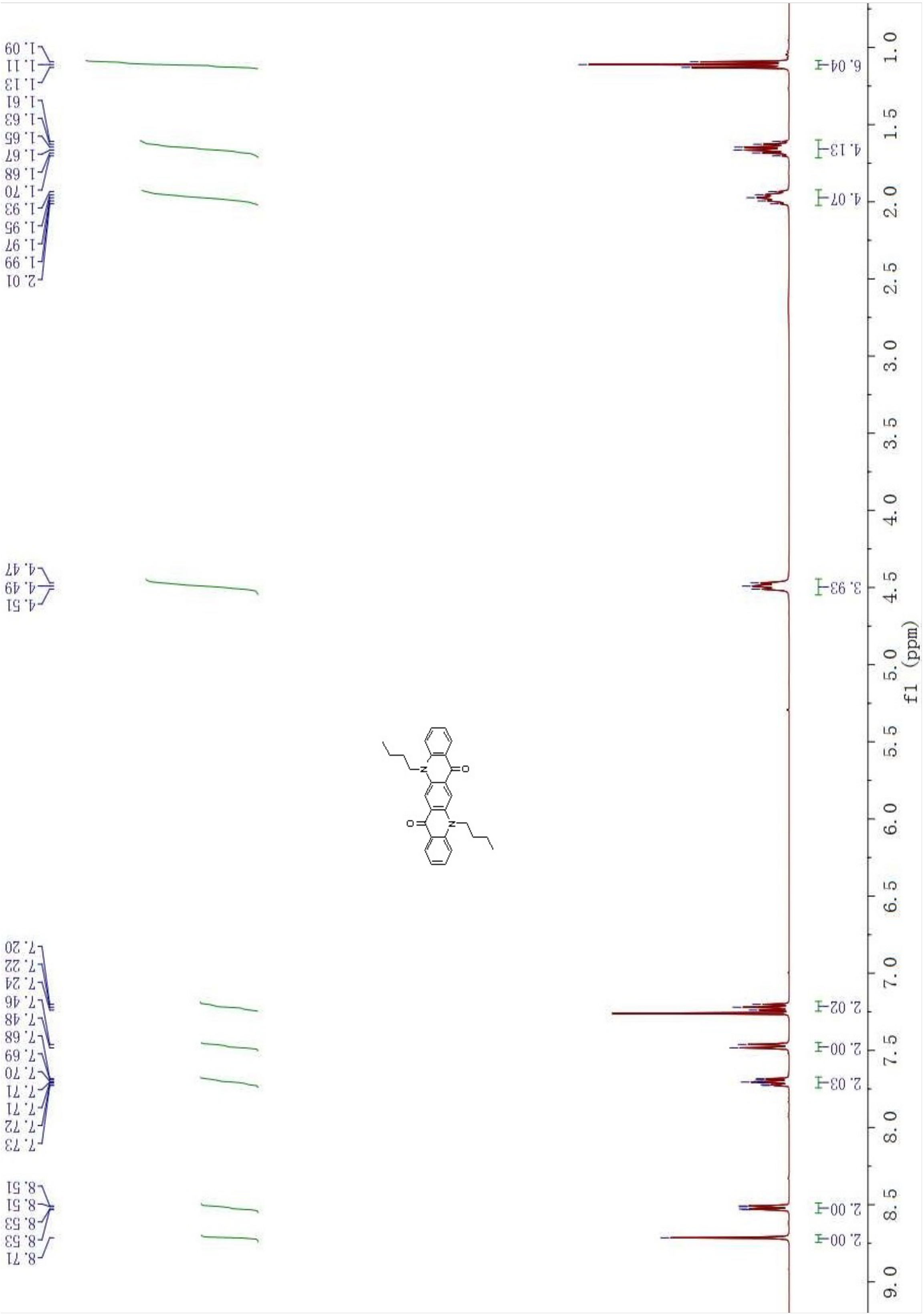

**NMR-7.** $^1$H NMR spectrum of compound **1** (400 MHz, $CDCl_3$).

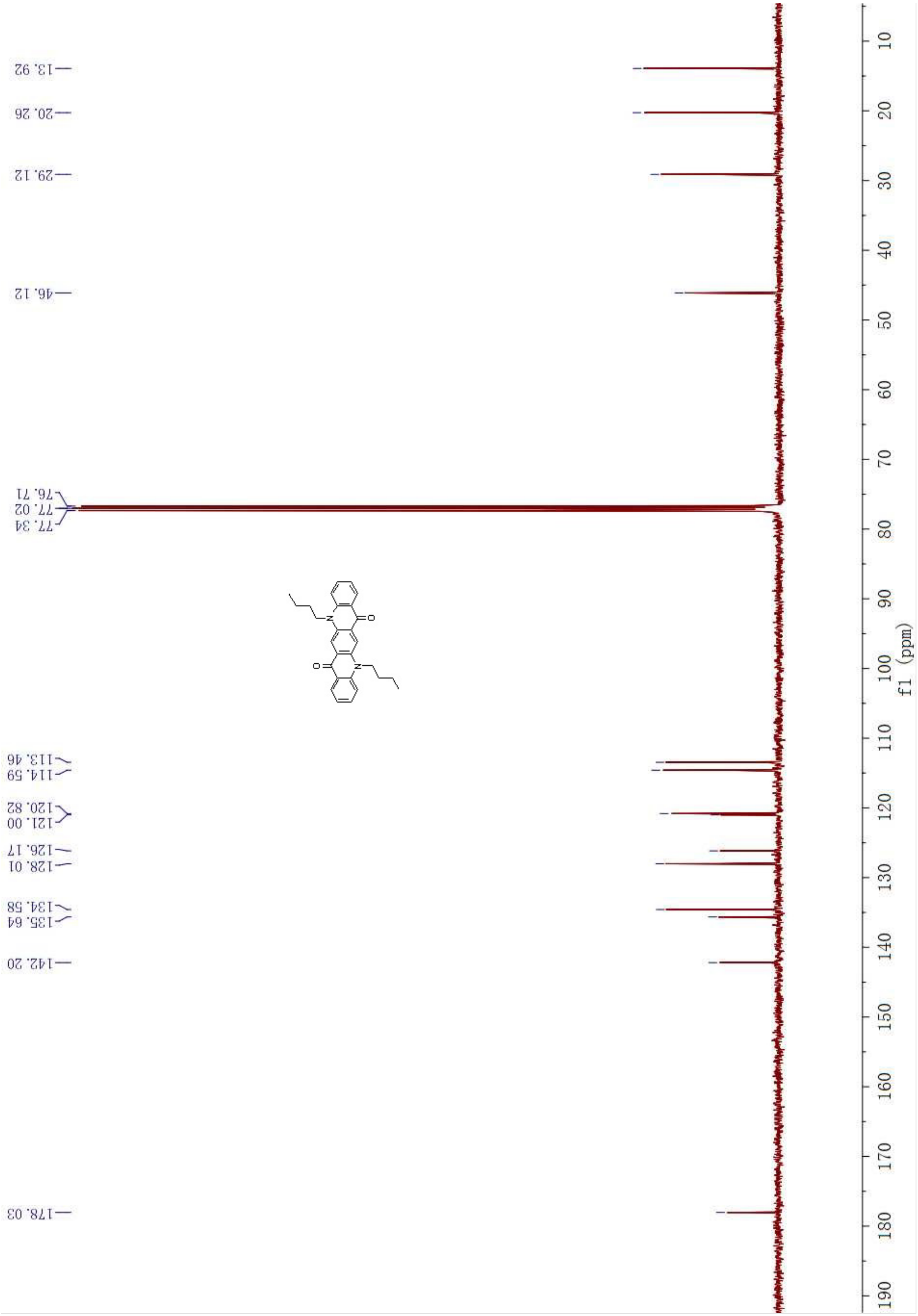


**NMR-8.** $^{13}C$ NMR spectrum of compound **1** (101 MHz, $CDCl_3$).
